# Gamma Delta T Cells in Shrimp Allergy Express a Unique Cytotoxic Cytokine Profile

**DOI:** 10.1002/clt2.70189

**Published:** 2026-07-30

**Authors:** Brenda Bin Su, Tyler Jackson, Warren Blackmon, Harold Ames, Christopher Holt, Aikaterini Anagnostou, Vibha Szafron, Sara Anvari, Hongjie Li, Carla M. Davis

**Affiliations:** ^1^ Immunology, Allergy, and Retrovirology Division of the Department of Pediatrics at Baylor College of Medicine William T. Shearer Center for Human Immunobiology Texas Children's Hospital Houston Texas USA; ^2^ Department of Molecular and Human Genetics Huffington Center on Aging Baylor College of Medicine Houston Texas USA; ^3^ Program in Cancer Cell Biology Baylor College of Medicine Houston Texas USA

**Keywords:** gamma delta T cells (γδT cells), shrimp allergy, single cell RNA sequencing, tropomyosin

## Abstract

Shellfish allergy is the most common food allergy in adults and the third most common in children. γδ T cells have been identified as playing a critical role in antigen tolerance in allergic diseases in mouse models. In humans, γδ T cells may play a regulatory role in peanut immunotherapy, and their role in shrimp allergy remains unclear. We hypothesized γδ T cells play a regulatory role in shrimp allergic disease. We performed single cell RNA sequencing (scRNAseq) on peripheral cells from shrimp allergic (SA) and healthy control (HC) subjects after stimulation with shrimp tropomyosin. This revealed a significant expansion of γδ T cells with three distinct clusters. One γδ T cell cluster predominated in SA, characterized as CD8+ with a cytotoxic expression profile. We found significant upregulation of TGF‐β1 and downregulation of IL‐7R in SA‐stimulated γδ T cells, and IL‐10RA expression in stimulated SA total PBMCs. γδ T cells may play a role in shrimp allergic disease through lymphocyte‐mediated cytotoxin signaling and cytokine‐mediated signaling pathways, including TGFβ‐1, IL7/TSLP‐IL7R, and IL10‐IL10R pathways.

## 1 Introduction

Food allergy has become increasingly prevalent in recent years. Approximately 6 million adults and 1 million children in the United States have symptoms consistent with shellfish allergy [1, 2]. The prevalence of this disease makes shellfish allergy the most common food allergy in adults and the third most common food allergy in children [3]. Shellfish allergy includes shrimp, crab, and lobster allergies, with shrimp being the most common [2, 4]. Shrimp allergy is a life‐threatening condition, frequently resulting in anaphylaxis, and approximately 50% of all shellfish‐allergic adults have visited an emergency room for severe reactions during their lifetime [4]. Current standard of care is strict allergen avoidance and epinephrine auto‐injector carriage for treatment in the event of accidental shrimp exposure. Unlike other common food allergens (i.e., peanut and tree nut allergies), there are gaps in our knowledge of the immunology of shrimp allergy, including the role of the major allergen contribution to immune responses, IgE antibody‐antigen epitope binding sites, diagnostic test specificity and sensitivity, and immunologic and clinical cross reactivity with other species [5, 6, 7, 8].

Shrimp allergy is typically triggered by specific proteins found in shrimp. The primary allergen responsible for allergic reactions to shrimp is tropomyosin (TM), a muscle protein [5, 6]. TM is also present in other shellfish, such as crab and lobster, so individuals allergic to shrimp may also react to these other crustaceans and mollusks [7, 8, 9, 10]. TM is the major allergen in shrimp, and there are multiple sequence alignments of TM from shrimp (Pen a 1, Pen m1) to crab (Por p 1), lobster (Hom a 1), and house dust mite (Der p 10) [11, 12].

Many cells are involved in food allergy, including peripheral immune cells such as mast cells, type 2 innate lymphoid cells, eosinophils, macrophages, and basophils, along with tissue epithelial cells [13]. T cells play a crucial role in orchestrating an adaptive immune response to food antigens. Antigen specific responses to food are typically mediated by T and B cells, whereas non‐specific responses are mediated by innate immune cells, like NK cells. T helper cells (Th2 cells) release cytokines that promote the production of antibodies, especially immunoglobulin E (IgE) produced by B cells, which bind effector cells to cause antigen specific allergic reactions [13, 14].

Previous research has highlighted the role of various cytokines (interleukin (IL)–1, IL1R (ST2), IL–4, IL–5, IL–9, IL–13, IL‐17A, as well as IL‐4 receptor pathways and thymic stromal lymphopoietin (TSLP) signaling) in the development of type 2 responses to food allergens [15]. Inhibitory cytokines have also been identified as IL–10, TGF‐b, and IFN‐γ. These pathways are well‐established in atopic conditions like allergic asthma and peanut allergy, however, their specific involvement in shrimp allergy remains unclear.

In food allergy, specific IgE (sIgE) antibodies bind to mast cells and basophils, sensitizing them to the allergen. Functional allergen‐specific T regulatory cells (Treg) play a very important role in food allergy to maintain homeostasis [16]. Treg cells contain a subset of cells with the ability to suppress allergic responses through the production of IL‐10 and TGF‐β cytokines. T regulatory mechanisms and these cytokines have been established as important in successful allergen immunotherapy [17].

Recent findings from our group revealed that gamma delta (γδ) T lymphocytes, including regulatory subsets (γδ Treg cells), may play a role in the modulation of oral immunotherapy (OIT) for peanut allergy, indicating their potential significance in desensitization processes [18]. They represent only 0.5%–5% of the total T cells in peripheral blood but much higher proportions in intestine (nearly 40%) and skin (10%–30%). In the context of shrimp allergy, a food allergy that elicits similar allergic responses to peanut, understanding the regulatory mechanisms of γδ T cells, including their impact on type 2 cytokine profiles and downregulation of regulatory functions, may unveil essential insight into the early consequences of immune stimulation with food allergens. We have observed through single cell sequencing in a peanut OIT trial that OIT causes differential regulatory T cell gene expression in memory and naïve γδ T cells within the first 6–24 weeks of therapy with OX40, GITR, TGFB1, CTLA4, ISG20 and CD69 specifically upregulated in memory γδ T cells [18]. The gastrointestinal tract reveals γδ T cell differential gene expression during peanut OIT, as further evidence for these cells in immune tolerance homeostasis to food [19]. Mouse models of egg allergy show tolerance to ovalbumin can be disrupted if γδ T cells are systemically removed, but the potential role for γδ T cells to promote oral tolerance has never been described in humans with shrimp allergy [20, 21].

Gamma delta (γδ) T lymphocytes are in the space between innate and adaptive immunity because they contain both CD8+ T‐cytotoxic and NK cell properties [22, 23]. γδ T cells have also been shown to be versatile T cells that can produce responses that have innate characteristics and also recognize antigens in a specific manner, but without the canonical need for major histocompatibility (MHC) antigen presentation. γδ T cells are primed to potently respond to pathogens and transform cells by recognizing a broad range of antigens. We found an increase in a subset of memory γδ T cells in the peripheral blood of some patients after specific food immunotherapy [18]. Human γδ T cells can exhibit an antigen‐presenting capacity. Like dendritic cells (DCs), blood Vγ9Vδ2 T cells are able to respond to signals from microbes and tumors and prime CD4^+^ and CD8^+^ T cells. Indeed, γδ T‐antigen presenting cells were also described to cross‐present antigens to CD8^+^ T cells [24, 25, 26, 27]. Several groups are now utilizing the cytotoxic properties of γδ T cells for anti‐tumor therapies [28, 29]. Their role in food allergic disease has yet to be fully elucidated, so demonstration of differential response after food‐antigen stimulation would extend our knowledge of their function.

In this study, we explored the role of γδ T cells in shrimp allergy through single cell RNA sequencing (scRNAseq) on peripheral cells from shrimp allergic (SA) and healthy control (HC) subjects after stimulation with shrimp tropomyosin [30, 31, 32, 33]. First, we describe the cellular composition of shrimp allergy compared to healthy peripheral blood samples, and then we show γδ T cell characteristics through gene expression, bead‐based flow cytometry, and functional pathway analysis. We evaluate the specific cytokine expression profiles of the γδ T cells after antigen stimulation and describe differences in cellular connections between immune cells in shrimp and healthy control peripheral blood. To our knowledge, this is the very first study to explore the role of γδ T cell immune responses to shrimp allergen in human cells.

## 2 Materials and Methods

### 2.1 Sample Characterization and Preparation and the Stimulation

Natural purified shrimp TM was purchased from Inbio (NA‐STM‐1, Charlottesville, VA. USA). All the samples were aliquoted and stored at −80°C until use. Cells were collected from Texas Children's Hospital (TCH) from shrimp allergic patients (SA, *n* = 16) and healthy controls (HC, *n* = 10) from 2016–2022. Each patient had experienced symptoms consistent with an IgE‐mediated reaction to shrimp in the past up to 10 years prior and had positive specific IgE to shrimp (f24) (Phadia 250 ImmunoCAP) (Thermo Fisher) or had a history of recent convincing repeated anaphylaxis episodes after eating shrimp. Healthy controls were able to eat shrimp without any allergic symptoms. Shrimp allergic subjects with positive shrimp IgE had a mean shrimp sIgE of 7.50 kU/L (0.5– > 100 kU/L) and the healthy controls all had shrimp sIgE of ≤ 0.3 kU/L. (Supporting Information S1: Figure S1) Institutional Review Board approval was obtained from Baylor College of Medicine, and informed consent was obtained for each subject (H‐39035). The samples were immediately centrifuged, and the separated plasma was aliquoted for storage at −30°C until later analysis.

Total peripheral blood mononuclear cells (PBMCs) were isolated by standard Ficoll‐Paque density gradient centrifugation and were frozen until use at −80°C. The frozen cells were rested overnight in RPMI‐1640 medium containing 2 mM glutamine, 10% heat‐inactivated human serum‐AB, and 1% penicillin/streptomycin in an incubator at 37°C and 5% CO2. These cells were stimulated with natural shrimp allergen TM at 1 μg/mL for 12 and 24 h if not indicated specifically. All subjects and parents provided written and informed assent and consent. This study was approved by the Institutional Review Board of the Baylor College of Medicine and TCH (H‐39035).

### 2.2 Single Cell RNA Sequencing (scRNAseq)

Cryopreserved PBMCs were rested in R10 media (RPMI‐1640, glutamine, 10% fetal bovine serum, 1% penicillin/streptomycin) followed by stimulation with 1 μg/mL shrimp TM for 24 h and then pooled together for single‐cell gene expression library construction. A cell viability of > 95% was required to advance to the single‐cell library construction steps (HC *n* = 3; SA *n* = 2). A subset of representative patient samples was utilized due to cost. The single‐cell gene expression library was prepared according to the Chromium Single Cell Gene Expression 5v2 kit (10x Genomics). In brief, single cells, reverse transcription reagents, Gel Beads containing barcoded oligonucleotides, and oil were loaded on a Chromium controller (10x Genomics) to generate single cell GEMS (Gel Beads‐In‐Emulsions) where full‐length cDNA was synthesized and barcoded. Subsequently, cDNA was amplified and fragmented to optimal size. The final library was sequenced on the Illumina NovaSeq 6000 platform, with a read length of paired end (PE) 150 bp (base pairs). Libraries were demultiplexed with Cell Ranger MKFASTQ and raw data was generated in FASTQ format. Raw FASTQ files were aligned using pre‐built human reference GRCh38 and processed with Cell Ranger version 6.1.2.

**FIGURE 1:**
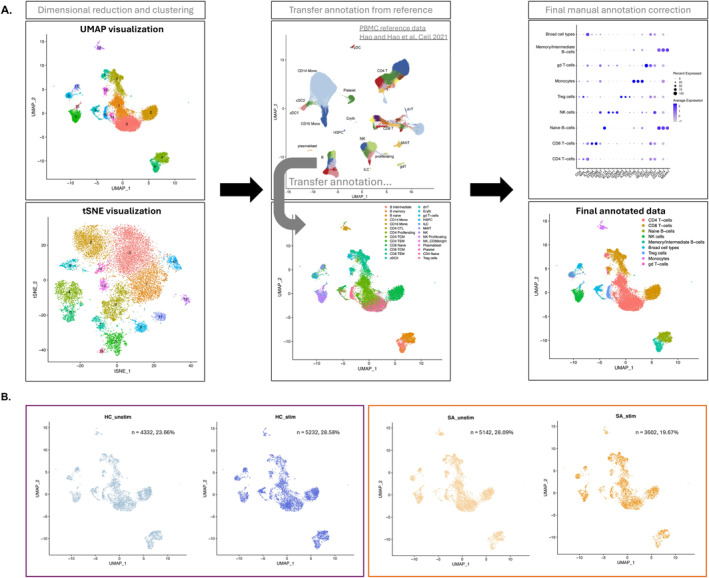
Annotation and clustering whole PBMC data. (A) Dimensional reduction, Leiden clustering, and annotation for the shrimp allergy PBMC scRNA‐seq data. The data was clustered after dimensional reduction. Annotation was conducted by transfer of a known PBMC reference from Hao and Hao et al., 2021 [34]. Lastly, the data was manually corrected using sets of marker genes for each cell type. (B) UMAP visualization of cell type changes between each sample group. Each plot contains the respective sample group's cell number from the original annotated data: Unstimulated HC (HC_unstim), stimulated HC (HC_stim), unstimulated SA (SA_unstim), and stimulated SA (SA_stim).

The .h5 matrix Cell Ranger output files were analyzed using the Seurat suite (version 4.3.0) implemented in R (version 4.2.2) [34]. (Supporting Information S1: Figure S2A) Ambient RNA was removed using SoupX followed by doublet removal using DoubletFinder. The individual sample Seurat objects were merged and integrated using Harmony, applying batch correction between different sample groups. The data were annotated using annotation transfer from the built‐in Azimuth PBMC (Figure 1A,B; Supporting Information S1: Figure S2B,C) [34]. The γδ T cells were annotated separately using a module score of 15 known marker genes (Supporting Information S1: Figures S3 and S4). Cells with a cutoff of > 0.5 were reannotated as γδ T cells. Downstream analysis such as differentially gene expression analysis, gene ontology analysis, and cell‐cell communication analysis were also performed. The differentially expressed genes (DEGs) between groups were calculated using the Wilcoxon rank‐sum test with Bonferroni correction. Given the low number of γδ T cells in the whole PBMC dataset, any gene with Log fold change > 0.5 was considered a DEG. DEGs were analyzed for enrichment of gene ontology terms related to Biological Process, Molecular Function, and Cellular Components. CellChat (Supporting Information S1: Figure S5) and NichNet (Supporting Information S1: Figures S6 and S7) analyses were implemented by conducting the cell‐cell communication analysis individually for each sample group and then comparing each result for differentially expressed pathways, according to the documentation on the GitHub repository [35].

### 2.3 Flow Cytometry

Total PBMCs from individual SA (*n* = 7) and HC (*n* = 8) were recovered overnight and stimulated with TM (1 μg/mL) for 24 h following by blocking and staining different cell surface markers. For the isolation of immune cell types of interest, the stimulated cells were washed and stained directly with titrated cocktails of fluorescently conjugated antibodies: monoclonal antibodies (mAbs) directed against CD3‐ AF700 (clone SK7), CD4‐ PE‐A (clone SK3), γδ TCR‐BV 421 (clone 11F2), CD16‐ BV480 (clone 3G8), Vδ2TCR‐BV510 (clone B6), CD28‐BV750 (clone CD28.2), CD62L‐BV785 (clone DREG‐56), αβTCR‐FITC (clone IP26), CD56‐PE/Cy5 (clone MEM‐188), CD27‐PE/Cy7 (clone O323), CD45RA‐AF700 (clone HI100), and CD3‐APC/AF750 (clone UCHT1) and with live/dead indicators like Zombie Dyes, and appropriate isotype controls were utilized and purchased from BioLegend (San Diego, CA, USA) and BD Biosciences, respectively. Non‐specific staining was prevented through FcBlock. The staining was performed in a total volume of 100 μl at room temperature (RT) for 30 min or at +4°C for longer followed by washing, resuspending the cells in 4% PFA/PBS, and loading into the flow machine. Cells were acquired on a LSRII Fortessa cytometer (BD Biosciences), and data were analyzed with FlowJo Software (Tree Star, Ashland, OR, USA) and with Kaluza Analysis Software (Beckman Coulter Life Sciences, Indianapolis, IN, USA). The flow compensation was conducted using the AbC total antibody compensation Bead kit (Invitrogen). Backgating analysis of the T γδ cell population was performed to determine the CD4 and CD8 positive γδ T cell proportions. (Supporting Information S1: Figure S8) Prism was utilized with *t*‐test and Fisher's exact tests to determine differences in cell populations.

## 3 Results

### 3.1 Peripheral Expansion of γδ T Cells in Response to Shrimp TM Stimulation

**FIGURE 2:**
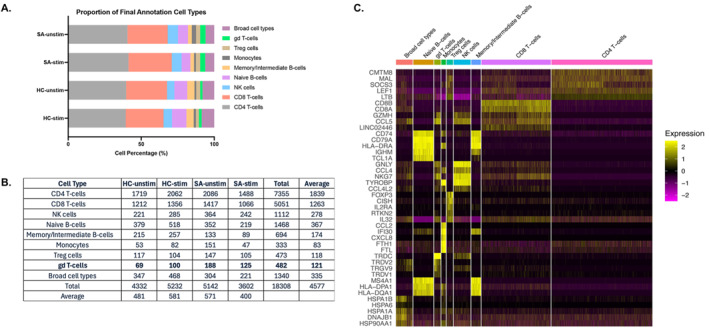
Peripheral γδ T cell expansion in SA in response to shrimp allergens and TM stimulation. (A) Bar plot displaying the percentage of cell types in each sample. (B) The table shows the absolute numbers of each cell type annotation by sample. (C) Heatmap showing the top 5 upregulated genes of each cell type annotation.

scRNAseq analysis and cellular annotation with marker genes were performed in healthy and shrimp allergic unstimulated and TM‐stimulated PBMCs (Figure 1A,B). This analysis revealed alteration in the cell population frequencies in response to shrimp allergen stimulation. There was a higher frequency of γδ T cells (*p* = 4.37E‐09) and lower frequency of memory/intermediate B cells (*p* = 5.07E‐09) in shrimp allergy (SA) patient unstimulated PBMCs compared to controls (Figure 2A and Supporting Information S1: Table S1). A total of 482 γδ T cells were detected in the PBMC dataset after processing with SoupX and DoubletFinder with an average of 121 cells in each sample SA versus HC (unstimulated and stimulated). The γδ T cells were significantly enriched in the SA sample compared to control in both unstimulated and TM stimulated comparisons (Figure 2B). Although not statistically significant, on stimulation, the absolute γδ T cell number changed from 68 to 100 in HC, but changed from 188 to 125 in SA. The heatmap reveals common genes shared between γδ T cells, NK and CD8+ cells, (GZMH, CCL4, CCL5, CCL4L2, GNLY, and NKG7) (Figure 2C.) showing that γδ T cells transcriptionally have a phenotype similar to NK and CD8+ cells.

**FIGURE 3:**
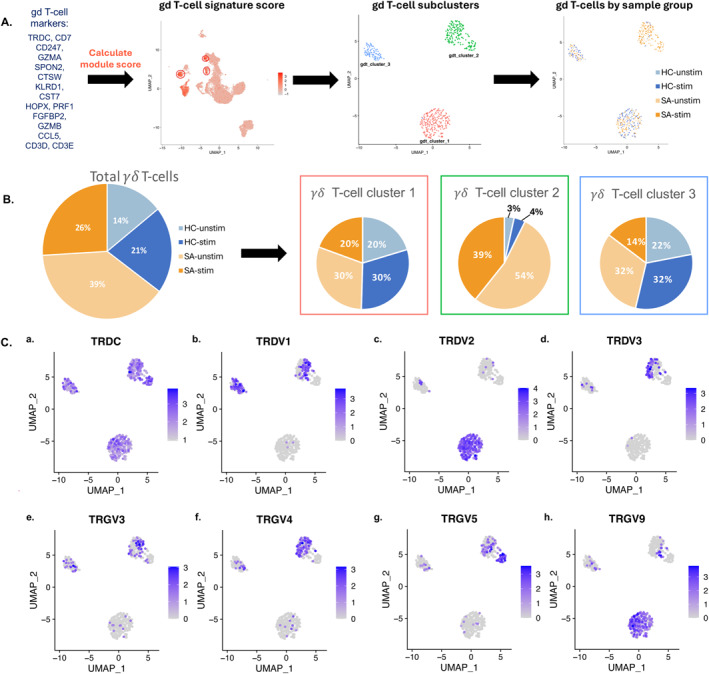
Characterization of γδ T‐cell Clusters in Shrimp Allergy. Figure 3 Characterization of γδ T‐cell Clusters in Shrimp Allergy. (A) We utilized the Seurat module score of several curated γδ T‐cell genes based on a rigorous search of γδ T‐cell related publications (PanglaoDB) [36]. 15 marker genes were used to generate the module score and cells that exceeded the > 0.5 module score threshold were annotated as γδ T‐cells. Three γδ T‐cell clusters were identified. (a), in whole PBMC data‐red circles; (b), cluster 1‐red, cluster 2‐green, cluster 3‐blue. γδ T‐cells were segregated into three distinct clusters and with the sample group displayed for each γδ T‐cell cluster. (shrimp—orange, healthy–blue) (B) Pie charts displaying the frequencies of γδ T‐cells in all the clusters combined and in each of the γδ T‐cell clusters. (C) γδ T‐cell TCR gene expression for each cluster. δ chain genes are plotted in the first row and the γ chain genes are plotted in the second row.

We utilized a more specific set of genes to annotate γδ T cells curated from the literature, which are shown in Figure 3A. Three clusters of γδ T cells were detected (Figure 3A), consisting of 39% γδ T cells from the unstimulated SA group, 26% γδ T cells from the stimulated SA group, 14% γδ T cells from the unstimulated HC group, and 21% γδ T cells from the stimulated HC group (Figure 3B). In cluster 1, there were a total of 222 γδ T cells, 178 γδ T cells in cluster 2, and 82 γδ T cells in cluster 3. The majority of γδ T cells in cluster 2 belonged to the SA group as opposed to the HC group. The γδ T cell proportions were as follows in cluster 2: unstimulated SA, 54%; stimulated SA, 39%; unstimulated HC, 3%; and 4% in stimulated HC. There was no difference of γδ T cells expansion in both clusters 1 and 3 among SA compared to HC.

**FIGURE 4:**
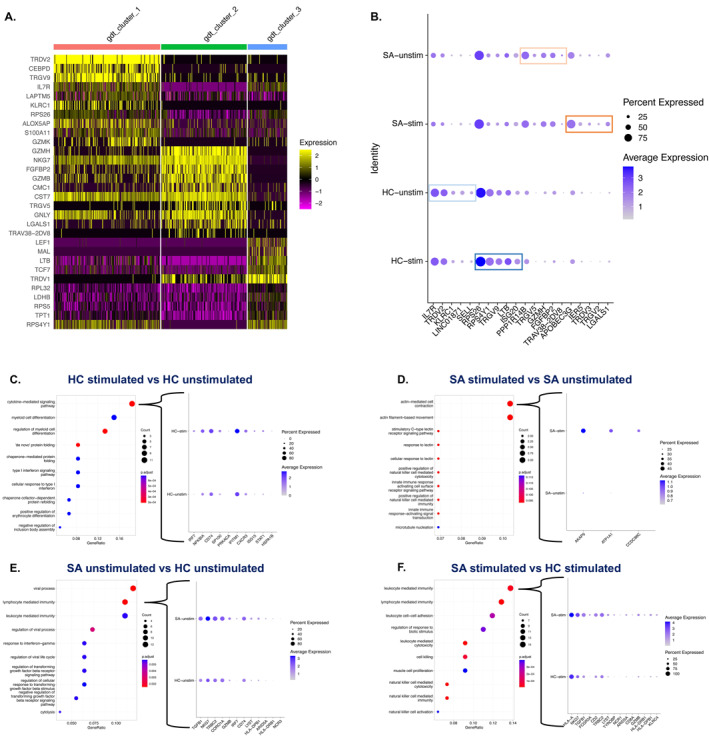
Percentage of γδ T‐cell Cell clusters in Healthy Control (HC) and Shrimp Allergy (SA) Samples by Flow Cytometric Analysis. (A) Increased γδ TCRhigh and TCRlow T cell populations in shrimp allergic patients from the pooled HC (*n* = 3) and SA (*n* = 2) after TM stimulation. The SA cells show a higher proportion of a γδ TCRhigh T cell population (2%). (B) γδ TCRhigh population in individual SA patients shows the SA γδ TCRhigh population is predominantly CD8+. (C) Vd1 and Vd2 staining reveals variable delta chain dimorphism within CD8+ cells. Vd2+ and TCR γδ cell populations are higher in SA compared to HC. (D–F) Gene ontology (GO) analysis between subclusters of γδ T‐cells and the cytokine profiles of the γδ T‐cells. Alteration of cytokines transcripts and secretion in response to shrimp allergens and shrimp TM. (D) The expression level of IL10RA in the whole PBMC data between each sample group. (E) The expression level of TGFB1 in the total γδ T‐cells for each sample group. (F) The expression level of IL7R in the total γδ T‐cells for each sample group. The adjusted *p*‐values are shown for the respective comparisons in the blue bars.

Cluster 1 was enriched for cells expressing TRDV2 and TRGV9 (Figure 4B), whereas cluster 2 was enriched for cells expressing TRDV1 and 3 and TRGV 3, 4, and 5 shown by scRNAseq (Figure 3C). Cluster 3 was enriched in TRDV1 and TRGV3 (Figure 3C). Cluster 2 has clear expression of Vd1 and Cluster 3 has clear expression of Vd2 in Supporting Information S1: Figure S11 by flow cytometric analysis.

### 3.2 Upregulation of γδ T Cell‐Cell Killing and Cytotoxin Signaling Pathways in SA, and Cytokine‐Mediated and Myeloid Differentiation in HC

The transcritional heatmap showed that unique gene expression between three clusters of γδ T cells as shown in Figure 5. Cluster 1 was enriched with TRDV2, TRGV9, CEBPD, IL7R, and GZMK transcripts, while cluster 2 was enriched with GZMH, GZMB, NKG7, GZMB, FGFBP2, and most of TRDV1 was found in cluster 2 and 3 (Figure 3C). We observed IL7/TSLP‐IL7R signaling pathways in γδ T cells, downregulation of IL7R gene transcripts in SA compared to HC samples (*p* adj = 0.005), and SA‐stimulated compared to HC stimulated (*p* adj = 0.007) (Figure 5B).

FIGURE 5Heatmap depicting the top 10 genes and gene ontology (GO) analysis. (A–B) Molecular differences within the subpopulations of γδ T‐cells. (A) Heatmap depicting the top 10 genes between the 3 subpopulations of γδ T‐cells. (B) Top 5 upregulated genes in the combined γδ T‐cell subpopulations by sample group. (C–F) GO analysis of the γδ T‐cells between different sample groups. EnrichR GO analysis was performed to obtain the top 10 Biological Process (BP) terms in the (C) HC‐stimulated versus HC‐unstimulated comparison with the accompanying upregulated genes in the top GO term. (D) SA‐stimulated versus SA‐unstimulated comparison with the accompanying upregulated genes in the top GO term. (E) SA‐unstimulated versus HC‐unstimulated comparison with the accompanying upregulated genes in the top GO term. (F) SA‐stimulated versus HC‐stimulated comparison with the accompanying upregulated genes in the top GO term. Genes selected for the GO term analysis were selected solely from a logfoldchange > 0.5 cutoff due to low cell number.

**Figure:**
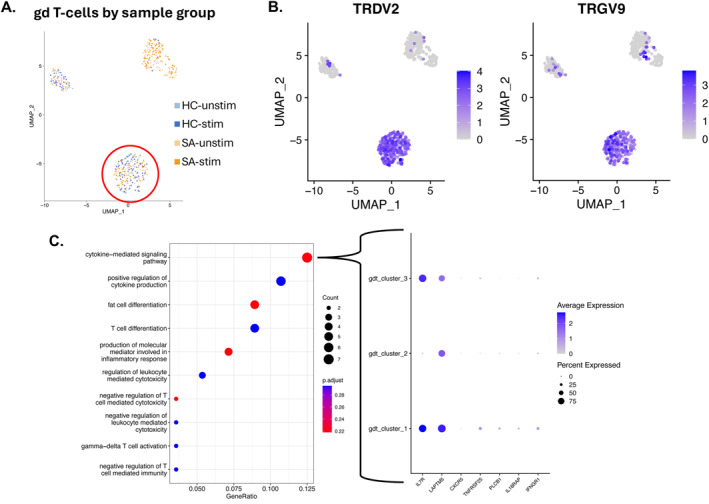

**Figure:**
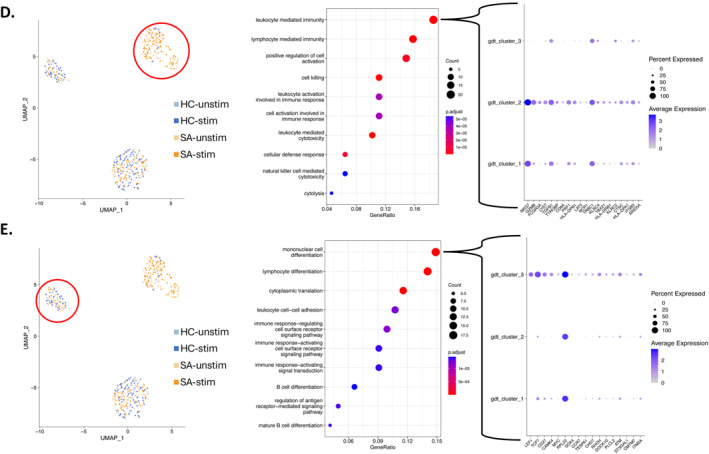

Other novel regulatory genes identified in γδ T cells in shrimp allergy include PP1 regulatory inhibitor subunit 14B (PPP1R14B), a protein with widespread expression which is a strong inhibitor of PP1, by acting on the ubiquitin–proteasome pathway. Gain‐ and loss‐of‐function assays have previously shown PPP1R14B promoted cell proliferation of triple‐negative breast cancer, colony formation, migration, invasion, and resistance to paclitaxel in vitro [30]. We found upregulated gene expression of PPP1R14B in SA as compared to HC in baseline (Figure 5B). Therefore, the ubiquitin‐proteasome pathway may play a role in food allergic disease.

EnrichR s showed upregulation of cytokine‐mediated and myeloid differentiation signaling pathways (padj = 0.0004) in γδ T cells from HC cells in response to TM stimulation including expression of IRF7 and NFkBA1 (*p* = 0.0002 and *p* = 0.0003, respectively) (Figure 5C). Upregulation of actin‐mediated cell contraction and actin filament‐based movement pathways in SA γδ T cells in response to TM stimulation (padj = 0.01) were characterized by differentially expressed upregulated genes in SA γδ T cells in response to TM stimulation (Figure 5D). We showed an upregulation of viral‐processing and lymphocyte‐mediated cytotoxic signaling pathways (padj = 0.0002) in SA unstimulated versus HC unstimulated γδ T cells (padj = 0.0015). This upregulation included increased expression of TGF‐ β 1 (padj = 9.8 × 10^−6^) and NKG7 (padj = 0.0002) (Figure 5E). Even when comparing SA stimulated to HC stimulated γδ T cells, TGF‐ β 1 and NKG7 were significantly upregulated (Figure 5F).

**FIGURE 6:**
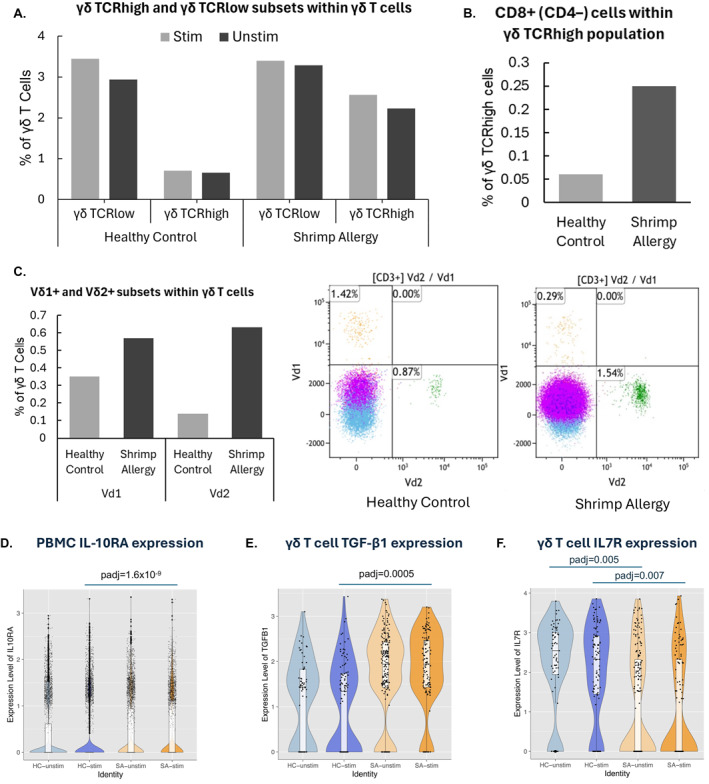
Gene ontology (GO) analysis of the γδ T‐cells between the γδ T‐cell clusters and the TRDV2/TRGV9 γδ T‐cells. (A) The cluster 1, TRDV2/TRGV9 cluster, is indicated by the red circle in the γδ T‐cell subcluster plot and the split by sample plot. (B) Marker genes TRDV2 and TRGV9 are plotted to highlight the TRDV2/TRGV9 γδ T‐cell subcluster. (C) EnrichR GO analysis was performed for γδ T‐cell cluster 2 versus the other two clusters. The top 10 upregulated Biological Process (BP) terms are shown with the accompanying upregulated genes in the top GO term. Genes used for the GO term analysis were selected solely from a logfoldchange > 0.5 cutoff due to low cell number. (D) UMAP highlighting γδ T‐cell cluster 2. The top 10 upregulated BP terms are shown with the accompanying genes from the top GO term for γδ T‐cell cluster two versus the other γδ T cell clusters. (E) UMAP highlighting γδ T‐cell cluster 3. The top 10 upregulated BP terms are shown with the accompanying genes from the top GO term for γδ T‐cell cluster three versus the other γδ T‐cell clusters. Genes selected for the GO term analysis were selected solely from a logfoldchange > 0.5 cutoff due to low cell number.

Since we identified three clusters of γδ T cells, we performed deep analysis of each cluster, shown in Figure 6, with cluster 1 characterized by TRDV2 and TRGV9 expression (Figure 6A–C). Cluster 2, the most enriched in SA, showed a higher gene expression of natural killer cell granule protein 7 (NKG7) padj = 3.13 × 10^−48^, granzyme B (GZMB) (padj = 3.08 × 10^−54^), and FCGR3A, also known as CD16a, (padj = 9.27 × 10^−33)^, respectively) (Figure 6D). Cluster 3, the smallest cluster, showed lymphoid enhancer‐binding factor 1 (LEF1) expression, a member of the family of the regulatory “T cell factor/LEF” family of proteins. (Figure 6E). Higher expression of IL7R (padj 4.69 × 10–30) was seen in both clusters 1 and 3.

Two molecularly distinct subsets of γδ T cells in SA and HC were found using antibodies against CD3+ and γδ TCR + by flow cytometry (Figure 4 and Supporting Information S1: Figure S11). There was an increased proportion of CD8+ γδ TCRhigh T cells within the γδ T‐cell population in SA compared to HC (2.23% in SA vs 0.65% in HC in unstimulated γδ T cells and 2.56% in SA vs 0.71% in HC in stimulated γδ T cells) (Figure 4A). We found a distinct γδ TCRhigh population in the SA group. (Figure 4B) In Figure 4B, we show that the γδ TCRhigh population in SA was predominantly CD+(CD4‐), representing 0.25% of γδ TCRhigh cells. While HC PBMC had little to no visible γδ TCRhigh population (0.06%). Additionally, the γδ TCRlow T cells in SA stained expressed Vd2, consistent with the cluster 3 phenotype noted by scRNAseq. Analysis of the γδ TCRhigh population revealed a higher percentage of CD8+ cells in SA as compared to HC. In Figure 4C, γδ T cells were identified in HC and SA by staining Vd1, Vd2, and γδTCR. SA samples showed a higher proportion of Vδ2+ cells within the γδ T‐cell population (gated from CD3+ T cells) compared to HC, whereas HC samples were relatively enriched for Vδ1+ cells. These findings highlight potential functional differences in SA γδ T cell responses compared to HC.

### 3.3 Regulatory Cytokine Alteration in Response to Shrimp Allergens and TM

**FIGURE 7:**
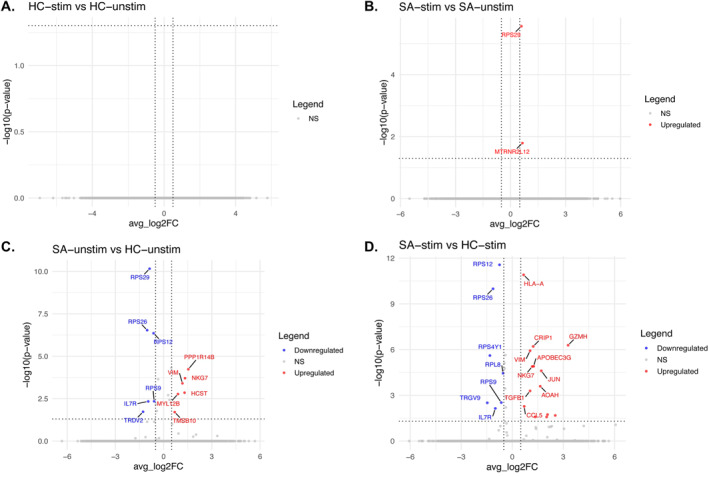
Volcano plots of differential gene expression (DEG) analysis between sample groups. The comparisons for each volcano plot are: (A) HC‐stim versus HC‐unstim, (B) SA‐stim versus SA‐unstim, (C) SA‐unstim versus HC‐unstim, and (D) SA‐stim versus HC‐.stim.

We next analyzed the cytokine transcripts from total PBMC and γδ T cells in response to shrimp TM stimulation. We found higher gene expression of IL‐10RA in the SA‐stimulated versus HC‐stimulated PBMCs (padj = 1.6 × 10^−9^) (Figure 4D) but the expression was not significant in γδ T cells. In γδ T cells, there was an upregulation of TGF‐β1 (padj = 0.0004) in SA versus HC in response to TM stimulation (Figure 4E) and downregulation of IL‐7R in baseline and SA‐stimulated versus HC‐stimulated (padj = 0.005, and padj = 0.007, respectively) (Figure 4F). We also determined GO pathways using FDR that are significant in the adjusted *p*‐value volcano plots in Figure 7 which show downregulation of IL‐7R, and upregulation of NKG7 and GZMH in SA versus HC.

### 3.4 Alteration of the Interaction of γδ T Cells With Other PBMCs

Cell‐cell communication analysis using CellChat identified γδ T cell communication at baseline and in response to TM stimulation to other immune cells is shown in Supporting Information S1: Figure S10. The direction of the signaling is shown with the arrows and the number of interactions depicted by the width of the arrows. In response to TM stimulation, the differential number of interactions in SA γδ T cells decreased from all cell types and was absent to Treg, CD4+ and CD8+ T cells (Supporting Information S1: Figure S10B) compared to the HC γδ T cells. (Supporting Information S1: Figure S10A) Interestingly, only monocyte interaction with γδ T cells was weakened in HC γδ T cells (blue color) upon stimulation. We observed an inverse effect of TM stimulation on the differential number of interactions to γδ T cells in SA (Supporting Information S1: Figure S10B). When comparing SA to HC at baseline (Supporting Information S1: Figure S10C), incoming signaling to γδ T cells (blue circle) some autologous communication with a larger number of differential interactions with NK cells and monocytes (shown as the thickness of the connection line) in SA cells as compared to T reg cells, memory B cells, naïve B cells, CD8^+^ T cells, and CD4^+^ T cells. Supporting Information S1: Figure S10A shows the strength of the incoming cellular interactions to HC γδ T cells increases with TM stimulation. Supporting Information S1: Figure S10B shows a slightly lowered interaction strength in SA γδ T cells upon TM stimulation, implying less γδ T function and γδ T Supporting Information S1: Figure S10C shows at baseline, γδ T cell incoming interaction strength of SA unstimulated cells is much higher than HC unstimulated cells.

## 4 Discussion

To our knowledge, this is the first study to explore the role of human γδ T cells in the pathogenesis of SA. We have employed a scRNAseq approach to reveal immune responses to natural shrimp TM antigen stimulation in SA and healthy human cell PBMCs and γδ T cells. Our studies reveal a potential regulatory role of γδ T cells in the shrimp allergen response. We have discovered a specific subset of γδ T cells unique to shrimp‐allergic subjects in both scRNA sequencing analysis and flow cytometry analysis. The SA γδ T cells have higher gene expression of TGF‐β1, but when PBMCs with γδ T cells were TM stimulated, there was lower expression of IL10RA and IL7R, indicating γδ T cells may play a regulatory role in food allergy pathogenesis.

γδ T cells are involved in the innate and adaptive immune system and mediate the immune response to pathogens and peanut allergens [18, 19]. In this study, we show peripheral expansion of γδ in response to shrimp allergens by both scRNAseq deep analysis and flow cytometry using a PBMC ex vivo experimental model, indicating γδ T cells may be involved in SA reactivity pathogenesis. This does not conclusively demonstrate antigen‐specific response, but does show differences in immune reactivity in SA versus HC cells. This γδ T cell population is enriched in SA, but contracts upon stimulation. In HCs, this population expands upon stimulation. Our hypothesis is that this may be indicative of the suppressive nature of these cells, ready to regulate in the normal state, but unable to control inflammation in the SA state.

This is the first time to show three clusters of γδ T cells with differential gene expression profiles that have been identified in food allergic disease (Figure 3). We found a higher frequency of γδ T cells in SA in baseline and in response to TM stimulation. Specific γδ T cell clusters 2 and 3 were enriched in SA cells and cluster 2 was characterized by expression of delta chain variable regions TRDV1 and 3 and gamma chain variable regions TRGV 3, 4 and 5. (Figure 3C). This could have implications for the antibody‐like recognition of antigens by γδ T cells, as structural analysis of the Vδ3 structure revealed close structural similarity to the VH domain of human IgM [37].

There is limited data regarding γδ T cell phenotypes in food allergic disease, especially from peripheral blood [38, 39, 40]. In mouse models of food allergy, a profound decrease in the percentage of γδ T cells in intestinal tissues and Peyer's Patches, but not in mesenteric lymph nodes or spleen, was found [41]. External gastrointestinal T cell receptor‐γδ(+) intraepithelial lymphocytes (IEL) have been shown to become activated selectively by cholera toxin to break oral tolerance in mice [38]. Intestinal γδ T cells modulate tissue responses to dietary nutrients by suppressing IL‐22 production, establishing a link between γδ T cells and intestinal adaptation to nutritional exposures. Intestinal γδ T cells are present in two anatomically and developmentally distinct tissue compartments: the IEL and lamina propria (LP) fractions with four transcriptionally distinct clusters. IEL γδ T cells have been described as having a cytotoxic phenotype, consistent with our findings. The LP γδ T cell fraction has shown to be enriched for genes found to be differentially regulated by diet, especially high carbohydrate diet [40]. Our data is consistent with the observation that IL7R is a differentially expressed gene in the LP γδ T cell fraction upregulated with diet exposure and that the IEL γδ T cell fraction is cytotoxic. Given the limited peripheral blood analysis of human γδ T cells in food allergic disease, the clusters we identified in our study may be most consistent with the previously described IEL and LP fractions. Indeed, a recent multi‐omic analysis of peanut oral immunotherapy identified enrichment of γδ T cells as indicative of the gastrointestinal regulatory role in treatment success [39]. Although γδ T cells have been shown to have context dependent functional plasticity, the regulatory role of γδ T cells is underexplored, and our findings implicate peripheral γδ T cells in food allergy regulation [39].

Vδ2 cells (TRDV2+) are known for rapid cytokine production, robust cytotoxicity, and responsiveness to phosphor‐antigen‐driven activation, features that may be amplified under inflammatory or pathogen‐driven conditions [37, 40, 41]. Vδ1 cells (TRDV1+) typically dominate tissue‐resident compartments and contribute to epithelial surveillance and homeostasis. The observed shift in γδ T‐cell subset composition between the disease and control groups suggests a disease‐associated remodeling of the γδ T‐cell compartment. Specifically, cells from SA exhibited a markedly higher proportion of Vδ2‐expressing cells, whereas HC samples were enriched for Vδ1‐expressing cells. This pattern indicates that the disease state may preferentially expand or recruit Vδ2 γδ T cells, potentially reflecting their functional specialization. Their increased abundance in the SA could therefore signify an enhanced innate‐like immune response or ongoing immune activation. Conversely, the relative reduction of Vδ1 cells in the disease is notable, as Vδ1 cells typically dominate tissue‐resident compartments and contribute to epithelial surveillance and homeostasis [40]. Lower frequencies in disease may indicate impaired barrier‐associated immunity or a redistribution of Vδ1 cells away from circulation. Together, these findings highlight disease‐specific alterations in γδ T‐cell subset dynamics in allergy and underscore the importance of dissecting functional differences between Vδ1 and Vδ2 populations to better understand their contributions to disease pathogenesis and immune regulation.

TGF‐β has been shown to be an important cytokine in food allergy, including Treg cell‐derived TGF‐β1 in regulating allergy, preventing food allergy and serving as one of the immunomodulatory factors regulating the development of tolerance [42, 43, 44]. It is possible that γδ T cells may attempt to rescue tolerance in food hypersensitivity by increasing TGF‐β1 expression in SA, but not in HC γδ T cells. This data supports the concept of a potential regulatory role for γδ T cells, showing TGF‐β1 expression increasing in γδ T cells in SA. A decrease in the expression of TGF‐β1 in regulatory T cells leads to an increase in the food allergic and autoimmune responses by disrupting oral tolerance‐promoting Treg cells, a process which can be rescued by the restoration of the expression of Treg cell‐derived TGF‐β1 [42].

Previous studies have demonstrated that IL‐7 can directly promote the survival and proliferation of activated human CD4+ T cells [45, 46]. Additionally, a clinical study demonstrated that IL‐7 can induce the expansion of human CD4+ T cells in vivo [47]. Lu et al., report that resting and activated CD4+ T cells expressed high levels of IL‐7Rα, but very low levels of TSLPR when compared with levels expressed in myeloid DCs, as was shown by Rochman et al. and human IL‐7 induces much stronger T cell activation, survival, and proliferation than TSLP [46, 48]. Bayer et al. demonstrated that IL‐7R signaling contributes to Treg cell development and peripheral homeostasis [49]. We found downregulation of IL‐7R transcripts in SA compared to HC γδ T cells, indicating shrimp allergic subjects have a deficiency in the regulation of TSLP/IL7‐ IL‐7R signaling pathway. This may also be one of the mechanisms of γδ T cell regulation in the pathogenesis of shrimp allergy.

IL‐10 (cytokine synthesis inhibitory factor) has multiple functions, such as inhibiting Th1 and Th2 activation and Th1/2 cytokine production, triggering B cells, granulocytes, and mast cells, keratinocyte growth differentiation, and NK cell proliferation and activation. Mutations of IL‐10 and IL‐10R can lead to severe dysregulation of the immune system. It is well‐known that IL10 plays an inhibitory function in food allergy, as it can promote regulatory T cell responses and decrease Th2‐dependent allergic responses. Though we found significantly lower IL‐10 secretion in SA versus HC total PBMCs in response to TM stimulation, there was a higher expression level of IL‐10R gene transcripts in SA, indicating IL‐10‐IL10R signaling plays an important role in SA [50].

Cellular communication analysis identified regulatory γδ T cell communication with Tregs, CD4+ and CD8+ T cells was interrupted in response to TM stimulation in SA, which may indicate loss of their positive regulatory effects. In the unstimulated state, γδ T SA cells had more differential number of interactions when compared to HC cells, showing γδ T cells are actively engaged in regulatory transcriptional efforts and increased strength compared to HC cells. We noted that γδ T cells are also engaged in autocrine communication, but further study is required to understand this effect. NK cells, memory/intermediate B cells, CD8+ T cells, and CD4+ cells clearly communicate with γδ T cells, which express three important TGF‐β, IL‐10, and IL7 signaling pathways (Figure 4). This is further evidence that γδ T cells are involved in the pathogenesis of SA.

In our transcriptional analysis, we found higher expression of GZMH and NKG7 in the SA specific cluster 2 which may explain the cytotoxic property of this γδ T cell subset (Figure 6A). It is not surprising that γδ T cells had similar patterns to NK cells in terms of upregulated genes in SA group in response to TM stimulation, indicating shrimp TM may have a similar molecular mechanism to activate γδ T cells as viral infection. NK cells have been found to be associated with peanut allergy and atopic dermatitis with invariant NK T cells stimulated by glycolipids in foods but have not been demonstrated in shrimp allergic disease [51, 52, 53]. Granzyme H, a member of the peptidase S1 family of serine proteases, plays a critical role in cytotoxic immune responses. Although traditionally associated with NK cells, where its constitutive expression and proteolytic activity contribute to alternative cell‐death pathways, accumulating evidence suggests that GZMH participates broadly in immunity [54].

In our study, γδ T cells from the SA displayed significantly higher levels of GZMH transcripts compared with those from HC, highlighting a disease‐associated shift toward a more cytotoxic phenotype. Enhanced GZMH expression within this cluster 2 suggests that γδ T cells may adopt an NK‐like cytotoxic program during disease, potentially contributing to food allergen clearance or, in some settings, tissue damage. Overall, the elevated GZMH transcription in γδ T cells in the SA not only reinforces their cytotoxic potential but also points to an underappreciated role for this subset in allergy disease pathogenesis. Further functional studies will be essential to determine whether GZMH‐high γδ T cells directly mediate protective food allergy [54]. In addition, NKG7 expressed by NK cells is critical for controlling cancer initiation, growth, and metastasis, indicating its role in immune homeostasis [55, 56].

### 4.1 Strengths and Limitations of the Study

This is the first study that aims to examine the cellular and molecular profile of γδ T cells from both SA and healthy peripheral blood in response to shrimp TM with assessment of gene and cytokine expression. Although γδ T cells are exceedingly rare in the peripheral blood, we have assessed both gene and protein expression of human γδ T cells with resulting insight. We found three clusters of differing characteristics and gene expression, which have not been described before in any food allergic condition. Our study has some limitations. First, a small sample size was used due to financial limitations, which may not capture all inter‐individual heterogeneity regarding SA. However, all cells were isolated from patients with evidence of IgE mediated symptoms and convincing anaphylaxis if IgE was low. Second, the depth of sequencing is also a limitation, as the γδ T cells only make up 0.5%–5% of all PBMCs and the number of cells sequenced was limited [5, 34]. The genetic and epigenetic backgrounds of subjects were not available, which may limit our understanding of any genetic predispositions and environmental factors that contribute to shrimp TM stimulation.

We do not know the underlying mechanisms and if this is a broader phenomenon in food allergy. We were not able to evaluate other food allergic cells, but this should be pursued in future studies. Additionally, since γδ T cells are found abundantly in the gastrointestinal tract, the peripheral γδ T cells may not reflect tissue‐based cell function. This will be important for future study. Differentially expressed transcripts from gastrointestinal gamma delta T cells were found to express key regulatory molecules such as IL‐10 and IL2RA (i.e., CD25) with some TGF‐β signaling pathway increased expression with immunotherapy treatment [19]. This is consistent with our findings in the periphery of significant upregulation of TGF‐β1 and downregulation of IL‐7R in SA‐stimulated γδ T cells, and decreased IL‐10RA expression in stimulated SA total PBMCs. However, we did not have access to tissue based γδ T cells, so we could not directly compare γδ T cells in both settings. Despite these limitations, our findings represent the differences between human SA γδ T cell response compared to healthy tolerant cells.

## 5 Conclusion

In conclusion, by using deep analysis of scRNAseq and bead‐based flow cytometric analysis, we have discovered that SA γδ T cells are enriched in peripheral blood of allergic subjects, have specific transcriptionally distinct clusters, and activate specific cytokine pathways in response to shrimp TM specific antigen stimulation. We found three distinct γδ T cell clusters. One SA γδ T cell cluster was characterized as CD8+ with a cytotoxic expression profile. We found γδ T cells likely play a regulatory role in SA through lymphocyte‐mediated cytotoxin signaling and cytokine‐mediated pathways. Human γδ T cells respond to specific antigen stimulation with TM with increased TGF‐β expression and downregulation of IL7R expression, potentially resulting in allergy through impairment of peripheral homeostasis and Treg development. This work expands the role of γδ T cells to include food specific allergic immune responses and further investigation into their potential regulatory role should be investigated in the future.

## Author Contributions

**Brenda Bin Su:** conceptualization, methodology, investigation, visualization, project administration, supervision, writing – original draft, writing – review and editing. **Tyler Jackson:** methodology, investigation, visualization, funding acquisition, project administration, writing – original draft, writing – review and editing. **Warren Blackmon:** methodology, investigation, visualization, project administration, writing – original draft, writing – review and editing. **Harold Ames:** writing – review and editing. **Christopher Holt:** visualization, project administration, writing – review and editing. **Aikaterini Anagnostou:** writing – review and editing. **Vibha Szafron:** writing – review and editing. **Sara Anvari:** writing – review and editing. **Hongjie Li:** conceptualization, funding acquisition, supervision, writing–review and editing. **Carla M. Davis:** conceptualization, methodology, visualization, funding acquisition, supervision, writing – original draft, writing – review and editing.

## Funding

NIH‐NIAID Grant #1R34AI57948. (C.M.D., B.B.S., W.B.) NIH‐NIAID Grant # U54 AI117804 (C.M.D., W.B., C.H.); NIH‐NIAID Grant # UM2‐AI130836 (C.M.D., W.B., C.H., S.A., H.L.); Regeneron Pharmaceuticals (C.M.D, S.A.); Takeda Pharmaceuticals (C.M.D, S.A.); NIH‐NIGMS Grant # T32GM136560 (T.J.); DBV Technologies (C.M.D, S.A.); Aimmune Therapeutics (A.A.), Novartis (A.A.); Genentech (A.A.), Bryn (A.A.); Baylor College of Medicine Pediatric Pilot Award (V.S.); CPRIT Scholar in Cancer Research (RR200063) (H.L.); Longevity Impetus Grant (H.L.); Ted Nash Long Life Foundation (H.L.); The Welch Foundation (H.L.); NIH/NIDDK 1F31DK141194‐01A1 (T.J.).

## Conflicts of Interest

The authors declare no conflicts of interest.

## Supporting information


Supporting Information S1

## Data Availability

All data are available in the main text or the supplementary materials.
