# Supplementary material for: Gamma Delta T Cells in Shrimp Allergy Express a Unique Cytotoxic Cytokine Profile
Source: Clin Transl Allergy. 2026 Jul 30;16(8):e70189. doi: 10.1002/clt2.70189 (PMC13425591; doi:10.1002/clt2.70189)
Supplement: Supplementary file 1 — Supporting Information S1 [file CLT2-16-e70189-s001.pdf]

Final Supplemental Figures

6_10_26

Supplemental Table 1: z-test of cell type proportions for each sample group comparison

Supplemental Table 1 - z-test of cell proportions

	CD4 T-cells	CD8 T-cells	NK cells	Na•ve B-cells	Memory/Intermediate B-cells	Monocytes	Treg cells	gd T-cells	Broad cell types
**HC-stim vs HC-unstim**	0.804173076	0.050174747	0.541562975	0.078701211	0.946445035	0.243909878	0.049918079	0.362391885	0.148183932
**SA-stim vs SA-unstim**	0.667893877	0.053132778	0.541562975	0.849867484	0.946445035	1.02E-06	0.928469142	0.687696493	0.698728317
**SA-unstim vs HC-unstim**	0.667893877	0.665521014	0.000325037	0.001253138	5.07E-09	4.7E-08	0.916158743	4.37E-09	0.000137359
**SA-stim vs HC-stim**	0.308914145	0.042319719	0.029920177	7.85E-07	1.72E-08	0.357425288	0.024073257	1.35E-05	6.39E-06

*** p-values are shown above for each of the respective comparisons. The two-sample proportion z-test was applied in each comparison. This was followed by a post ad-hoc Bonferroni-Hochberg correction to adjust for multiple comparisons**

Shrimp Allergy	Respiratory/Tongue/Throat Itching	Pruritic Rash/Urticaria	GI	Eye/Nasal	Neuro	Shock/CV	Anaphylaxis	Shrimp Specific IgE	Total IgE
**1**	N	Y	Y	Y	N	N	**Y**	0.46	274
**2**	N	Y	N	N	N	N	N	>100	1512
**3**	Y	Y	N	Y	N	N	**Y**	< 0.1	166
**4**	Y	N	N	N	N	N	N	0.5	255
**5**	Y	Y	N	Y	N	N	**Y**	< 0.1	107
**6**	N	N	Y	N	N	N	N	0.27	62
**7**	Y	N	N	Y	N	N	**Y**	0.88	942
**8**	Y	Y	N	Y	N	N	**Y**	0.52	847
**9**	N	Y	Y	Y	N	N	**Y**	66.4	1227
**10**	N	Y	N	N	N	N	N	1.62	799
**11**	Y	Y	Y	Y	N	N	**Y**	0.9	139
**12**	Y	Y	N	N	N	N	**Y**	0.5	85
**13**	Y	N	N	N	N	N	N	19.2	450
**14**	N	Y	N	N	Y	N	**Y**	<.10	34.6
**15**	Y	Y	N	N	N	N	**Y**	1.09	338
**16**	Y	Y	Y	N	N	N	**Y**	<.10	5.76
**Healthy Control**									
**1**	N	N	N	N	N	N	N	0.3	2311
**2**	N	N	N	N	N	N	N	< 0.1	141
**3**	N	N	N	N	N	N	N	<0.1	15
**4**	N	N	N	N	N	N	N	< 0.1	51.2
**5**	N	N	N	N	N	N	N	<0.1	90.7
**6**	N	N	N	N	N	N	N	<0.1	30.1
**7**	N	N	N	N	N	N	N	0.17	252
**8**	N	N	N	N	N	N	N	<0.1	30.6
**9**	N	N	N	N	N	N	N	<0.1	67
**10**	N	N	N	N	N	N	N	<0.1	75

Supplemental Figure 1. Shrimp allergy and healthy control donor characteristics.

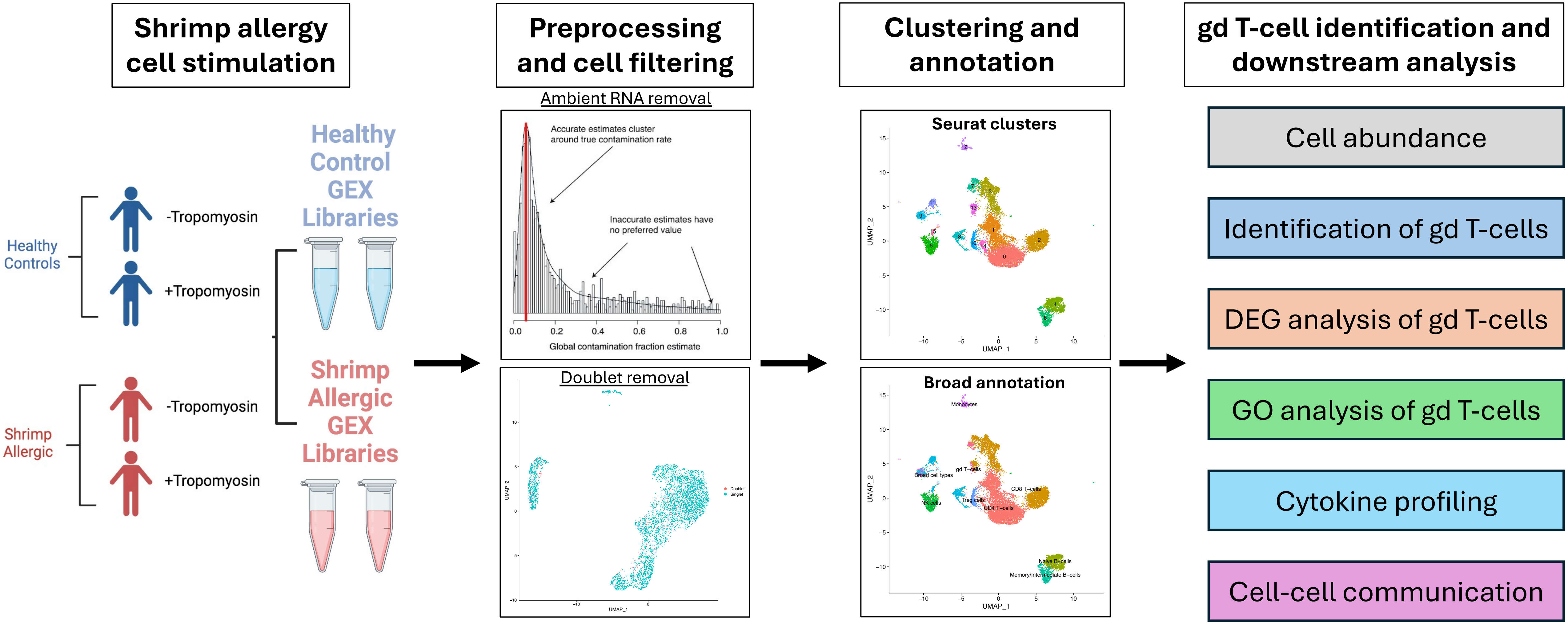

Supplemental Figure 2. scRNA-seq and downstream analysis pipeline of the whole PBMC data. (A) Pipeline for scRNA-seq and data analysis of the PBMCs from healthy controls (HC) (n=3) and shrimp allergic (SA) patients (n=2). The data was clustered using the conventional Seurat pipeline with 50 principal components for UMAP and tSNE plot generation (See Methods). This was then integrated with an existing PBMC reference using Azimuth. The downstream analysis included whole PBMC cell abundance differences, identification of γδ T-cells, DEG analysis, GO analysis, cytokine profiling, and cell-cell communication analysis

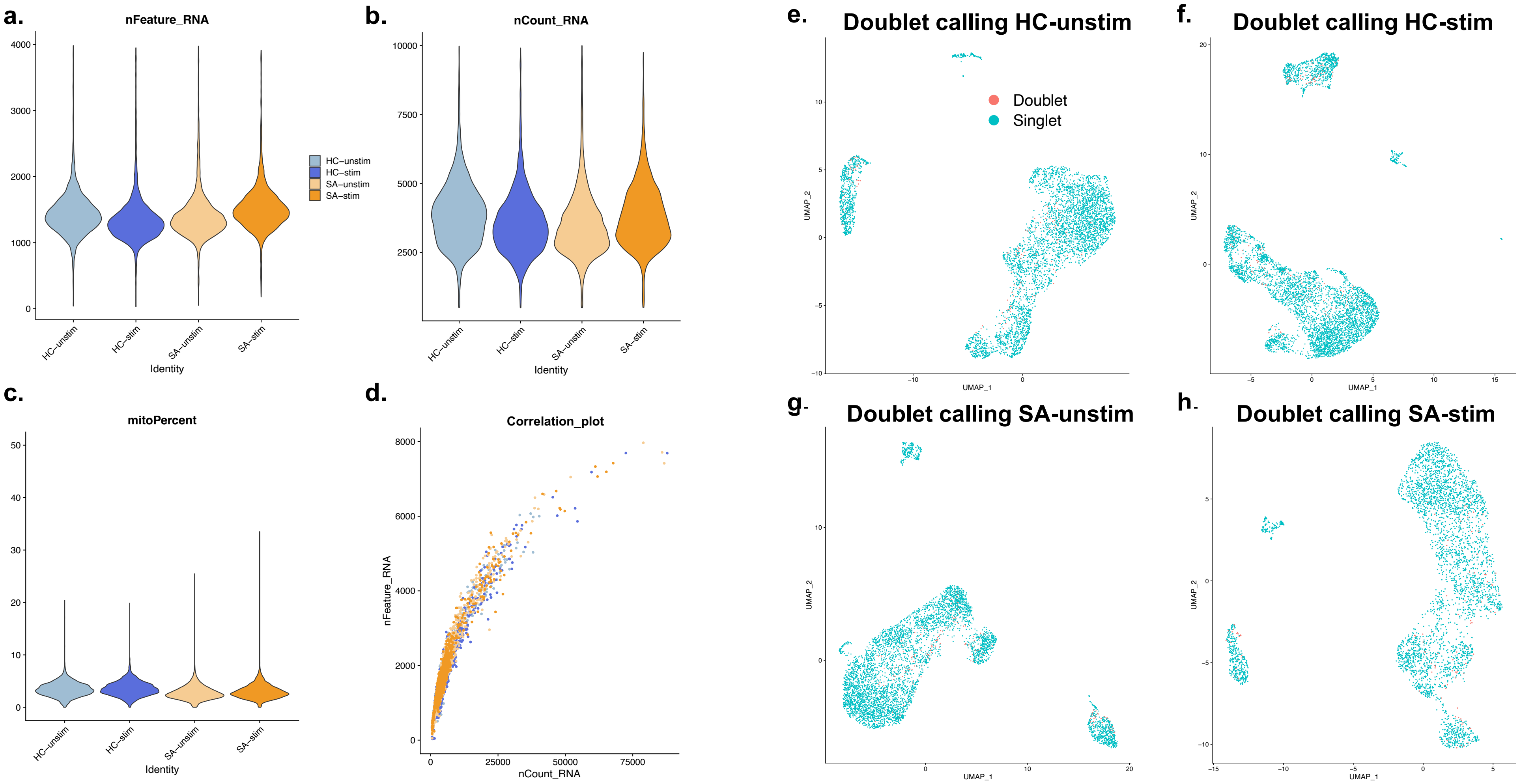

Supplemental Figure 2B. Quality control of the whole PBMC data. (B) Quality control metrics for the data before filtering. The (a) number of genes per cell, (b) number of transcripts per cell, (c) mitochondrial percent of counts per cell, and (d) a correlation plot for the number of genes vs number of transcripts per cell is shown. Results of the DoubletFinder doublet removal algorithm for (e) HC-unstim, (f) HC-stim, (g) SA-unstim, and (h) SA-stim. A total of 599 doublets were removed from the data.

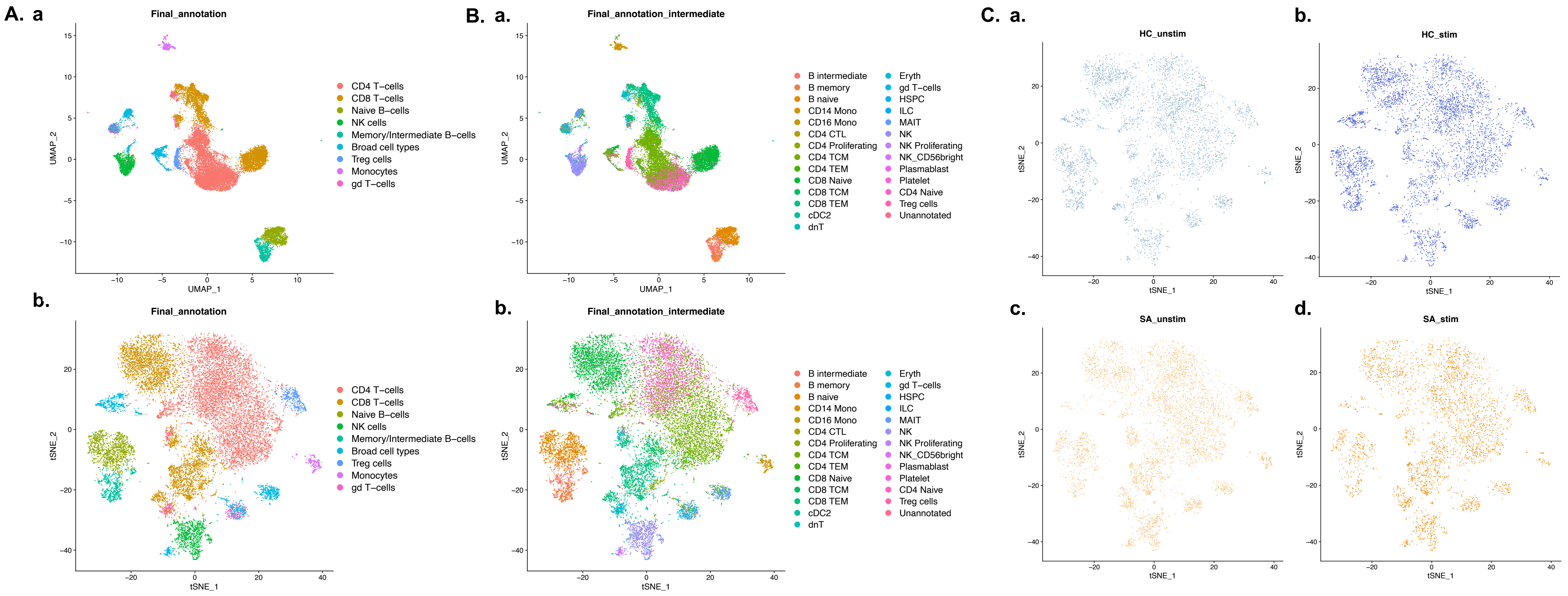

Supplemental Figure 2C. Whole PBMC clustering and annotation. (A). Broad annotation UMAP and tSNE for the pooled whole PBMC data. (a) UMAP with the broad annotation legend to the right. (b) tSNE with the broad annotation legend to the right. **(B).** Intermediate annotation UMAP and tSNE of the pooled whole PBMC data. (a) UMAP with the intermediate annotation legend to the right. (b) tSNE with the intermediate annotation legend to the right. **(C).** tSNE of the whole PBMC data split by sample group. The colors correspond to the sample group source in (a), (b), (c), and (d).

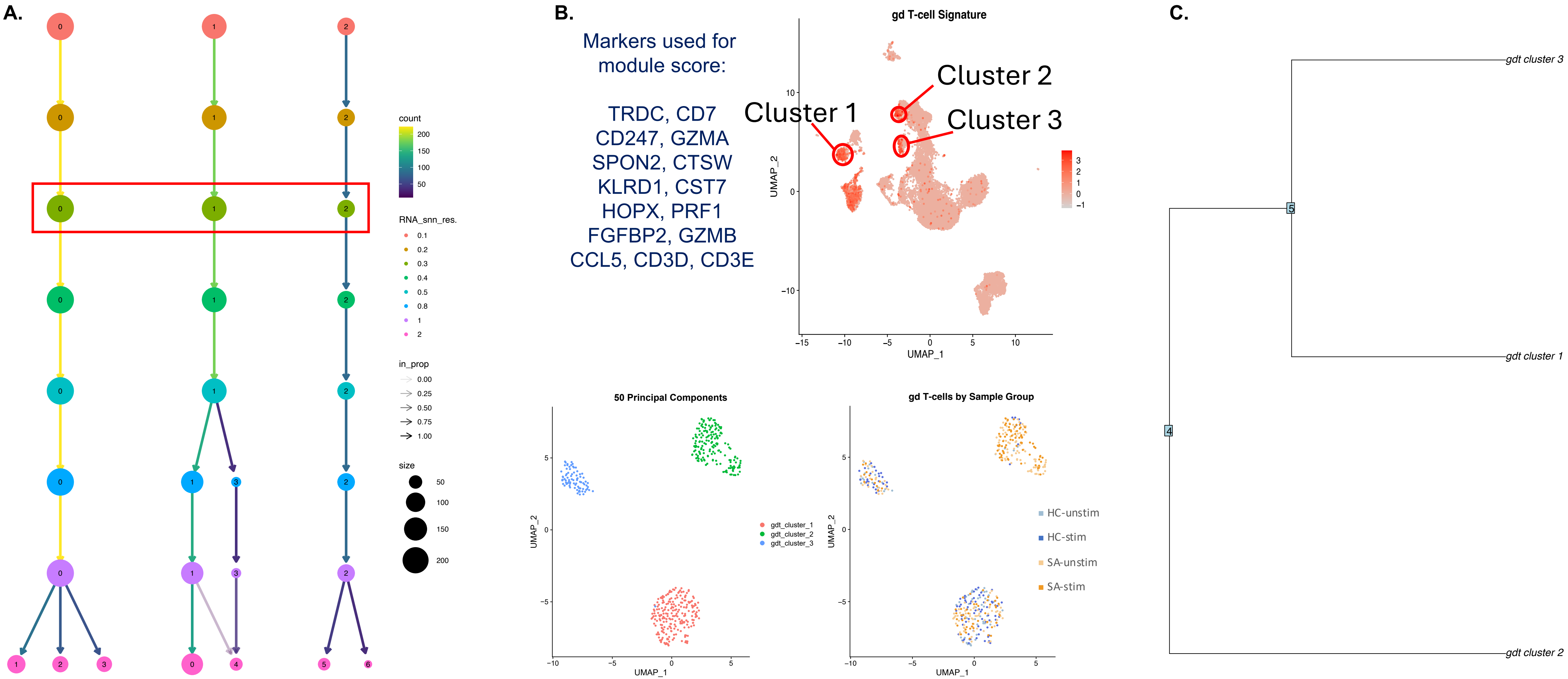

Supplemental Figure 3. γδ T-cell subcluster annotation and module score strategy. (A) Clustree analysis showing how many clusters each resolution contains. The most consistent number of clusters was three and therefore the 0.3 resolution was selected for clustering the γδ T-cells. **(B)** The γδ T-cells were selected from publication-curated markers from the PanglaoDB database for the γδ T-cells. The three clusters from the whole PBMC data are shown in the module score plot. These three annotated clusters with the different sample groups are shown in the UMAPs below. **(C)** Dendrogram for the three γδ T-cell subclusters to show how related they are based on transcriptomic profile.

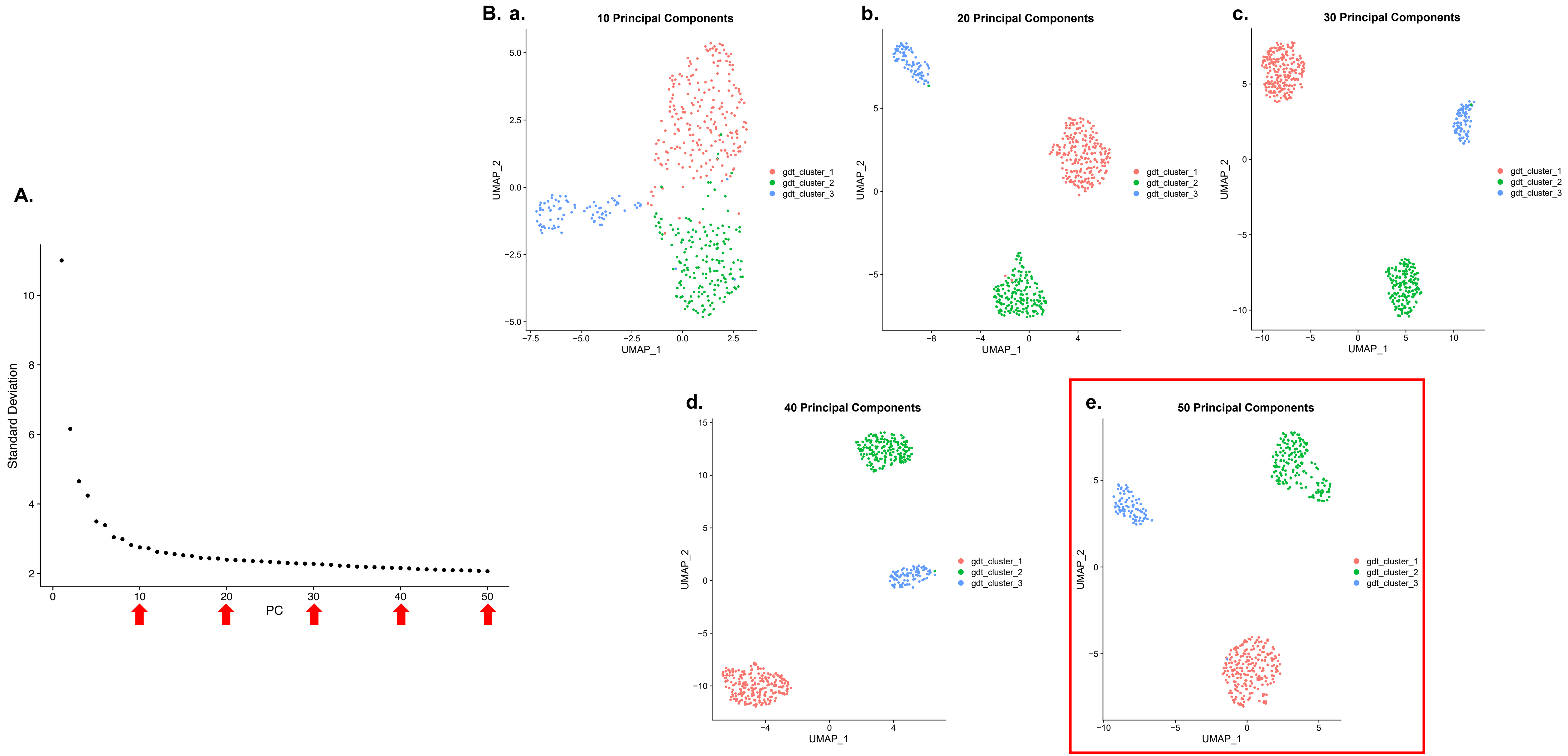

Supplemental Figure 4. γδ T-cell subclustering. (A) Elbow plot displaying the variation of explained by each principal component (PC) for the γδ T-cells. Each of the arrows indicate which PCs were used for subclustering. **(B)** UMAP graphs with the indicated PCs used for clustering from (A): (a) 10 PCs, (b) 20 PCs, (c) 30 PCs, (d) 40 PCs, (e) 50 PCs. 50 PCs were selected for downstream analysis as each of the other parameters preserved the same separation.

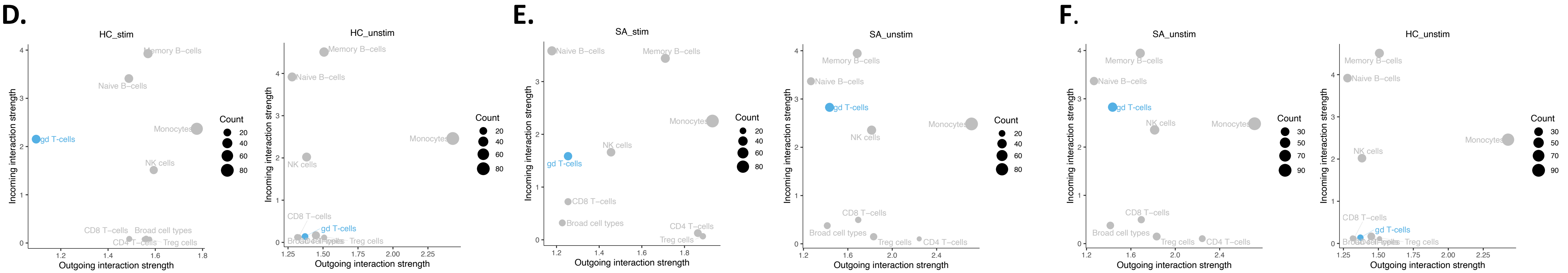

Supplemental Figure 5. CellChat cell-cell communication analysis of the cell-cell interactions in PBMC in response to shrimp allergens and to TM stimulation. (D-F) Signaling graphs showing the total incoming and outgoing signals in γδ T-cells between **(D)** HC stim vs HC unstim, **(E)** SA-stim vs SA-unstim and **(F)** SA-unstim vs HC-unstim. All genes were differentially expressed with p-values < 0.05 for each respective comparison.

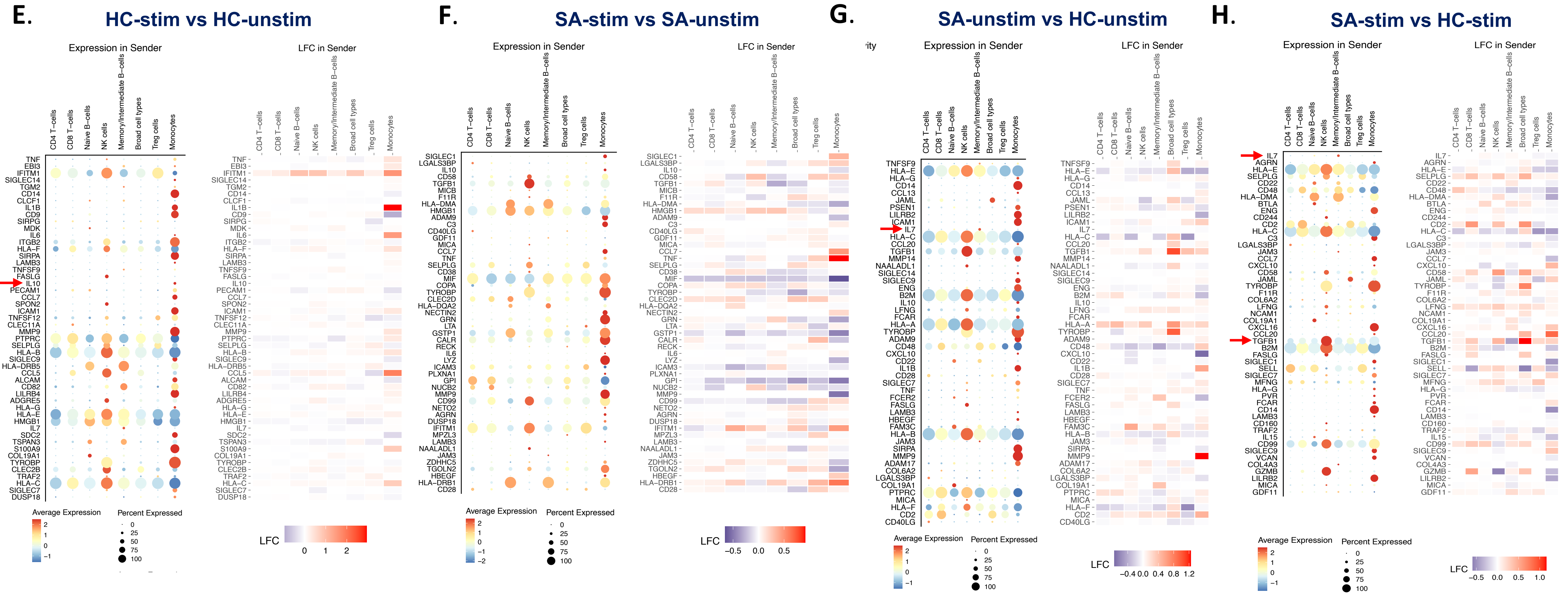

Supplemental Figure 6. NicheNet cell-cell communication analysis between gd T-cells for healthy controls and shrimp allergic patients. (D-G) NicheNet outputs for the **(D)** HC-stim vs HC-unstim, **(E)** SA-stim vs SA-unstim, **(F)** SA-unstim vs HC-unstim, **(G)** and SA-stim vs HC-stim comparisons. The expression of the respective genes in the sender cell is shown in the left panel and the logfoldchange of those sender genes between the respective comparisons is shown in the right panel for each comparison. The differentially expressed signaling genes were obtained by the Wilcoxon Rank Sum Test ($p < 0.05$). Observations are that IL10 is increased in HC-stim but not SA-stim, which supports the sequencing data. SA-stim vs HC-stim shows the upregulation of TGFB1 signaling as in sequencing data. IL7 signaling is also reduced in both SA-unstim vs HC-unstim and SA-stim vs HC-stim comparisons.

Supplemental Figure 7A. NicheNet : HC-stim vs HC-unstim

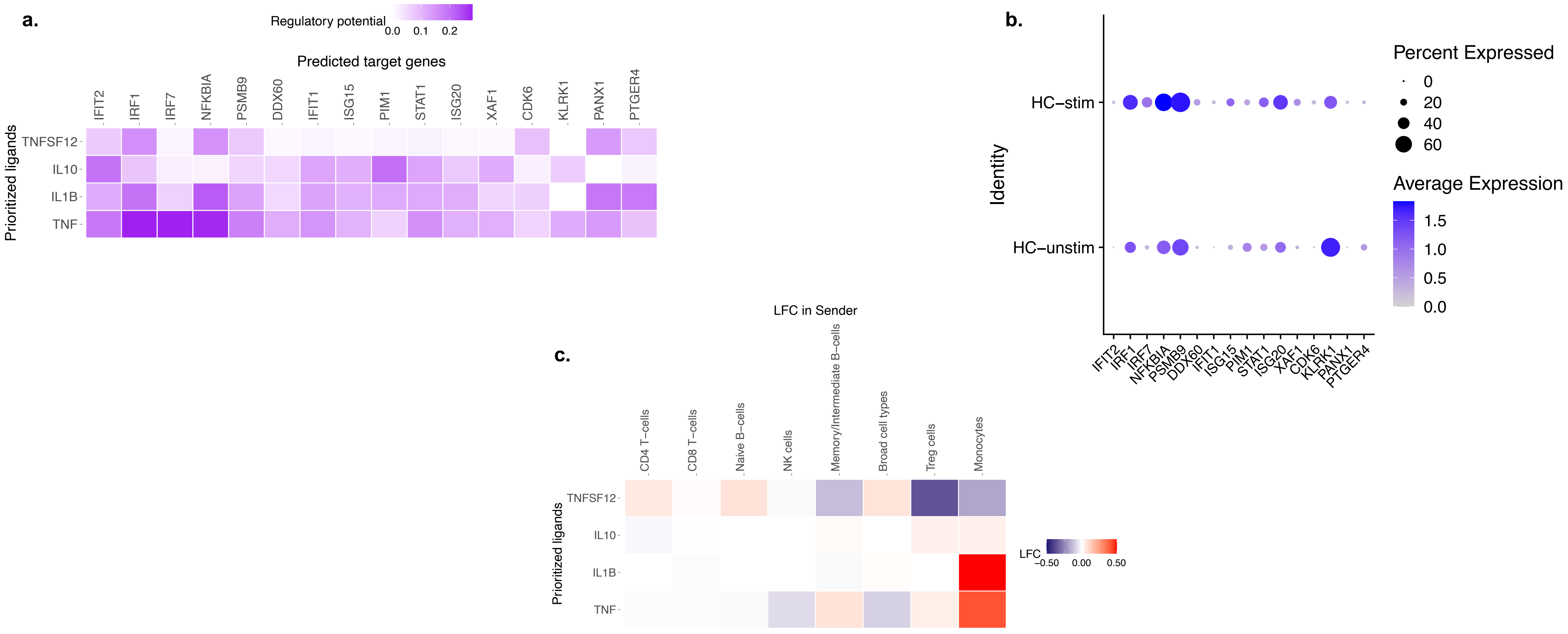

Supplemental Figure 7A. NicheNet cell-cell communication analysis of ligands and receptors in the HC-stim vs HC-unstim comparison. (a) Ligand and target gene analysis showing the top expressed ligands (rows) with their inferred target genes (columns). (b) Dot plot showing expression levels of the target genes from (a) between the HC-stim and HC-unstimulated sample groups. (c) Log fold changes of the ligands (rows) in each sender cell type (columns) between HC-stim and HC-unstim.

Supplemental Figure 7B. NicheNet : SA-stim vs SA-unstim

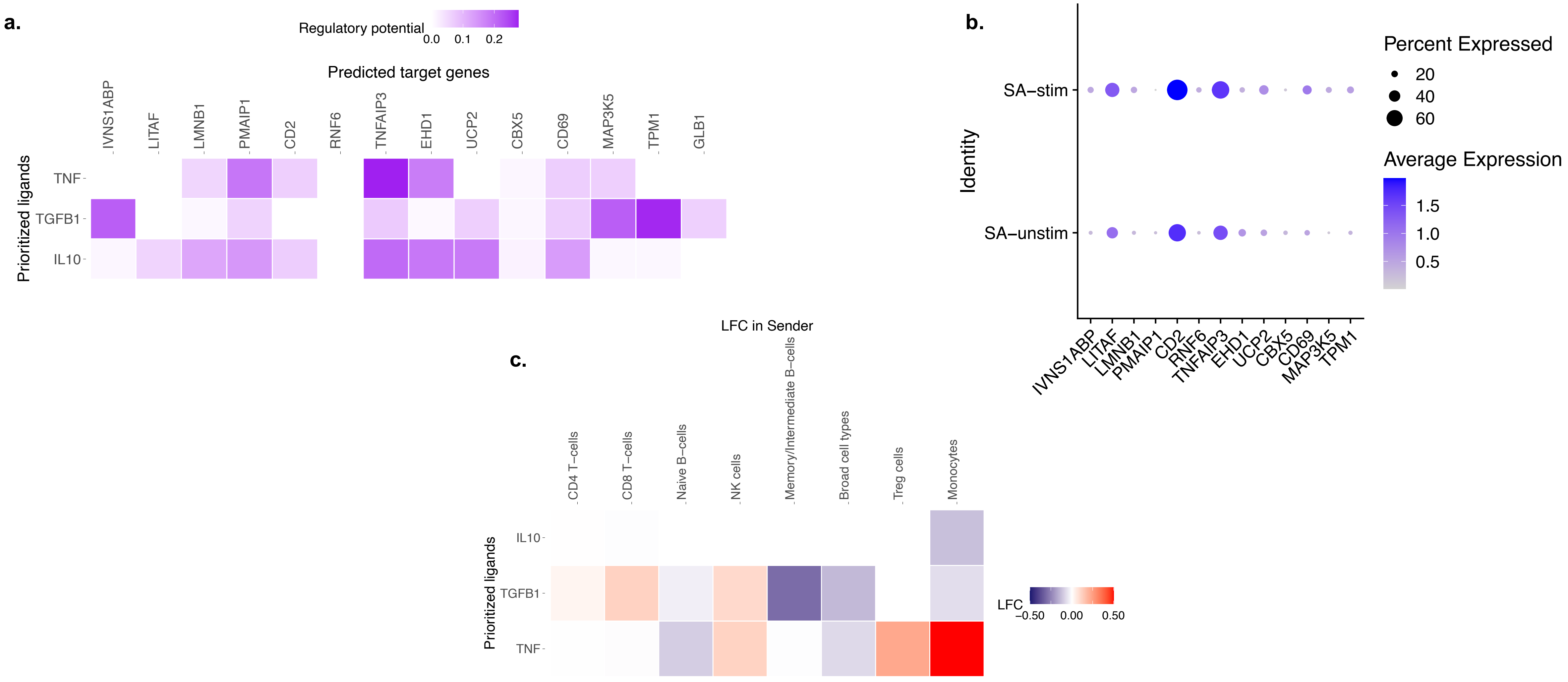

Supplemental Figure 7B. NicheNet cell-cell communication analysis of ligands and receptors in the SA-stim vs SA-unstim comparison. (a) Ligand and target gene analysis showing the top expressed ligands (rows) with their inferred target genes (columns). (b) Dotplot showing expression levels of the target genes from (a) between the SA-stim and SA-unstim sample groups. (c) Log fold changes of the ligands (rows) in each sender cell type (columns) between SA-stim and SA-unstim.

Supplemental Figure 7C. NicheNet : SA-unstim vs HC-unstim

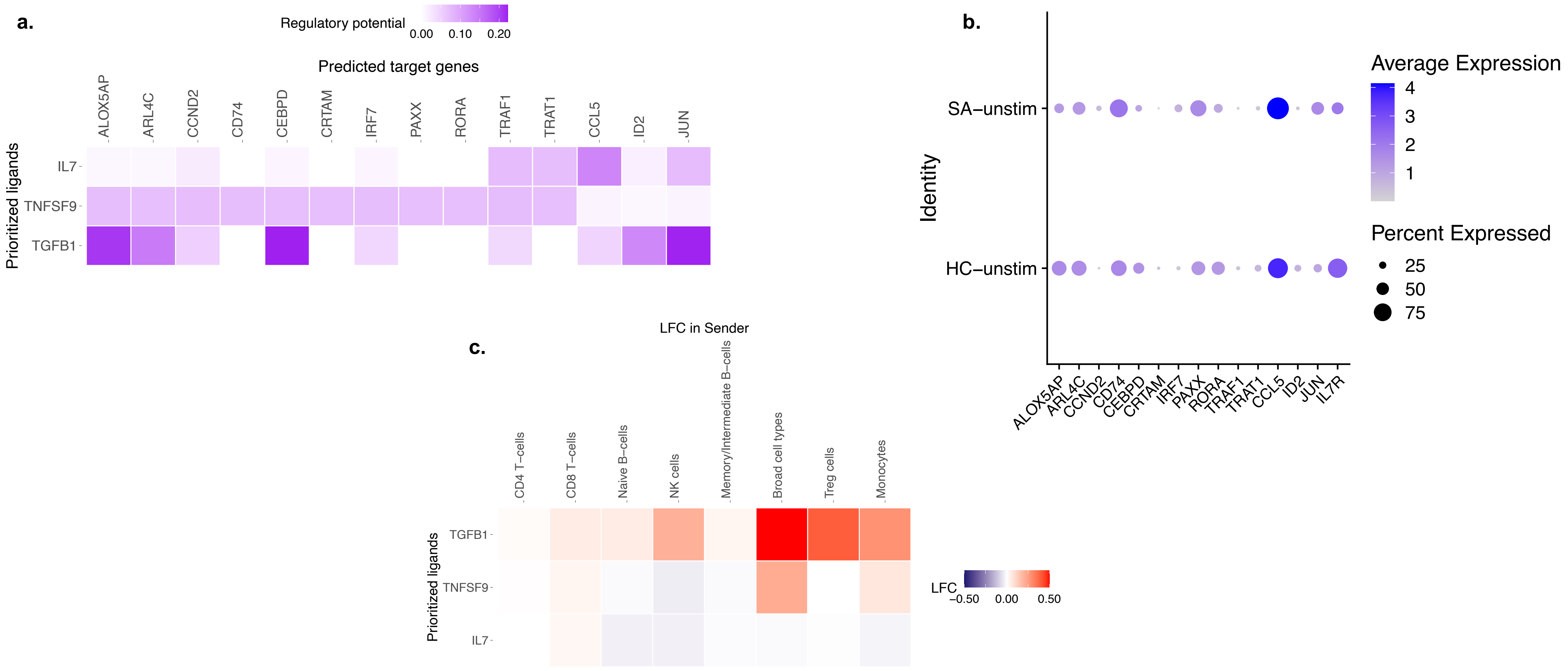

Supplemental Figure 7C. NicheNet cell-cell communication analysis of ligands and receptors in the SA-unstim vs HC-unstim comparison. (a) Ligand and target gene analysis showing the top expressed ligands (rows) with their inferred target genes (columns). (b) Dot plot showing expression levels of the target genes from (a) between the SA-unstim and HC-unstim sample groups. (c) Log fold changes of the ligands (rows) in each sender cell type (columns) between SA-unstim and HC-unstim.

Supplemental 7D. NicheNet : SA-stim vs HC-stim

a.

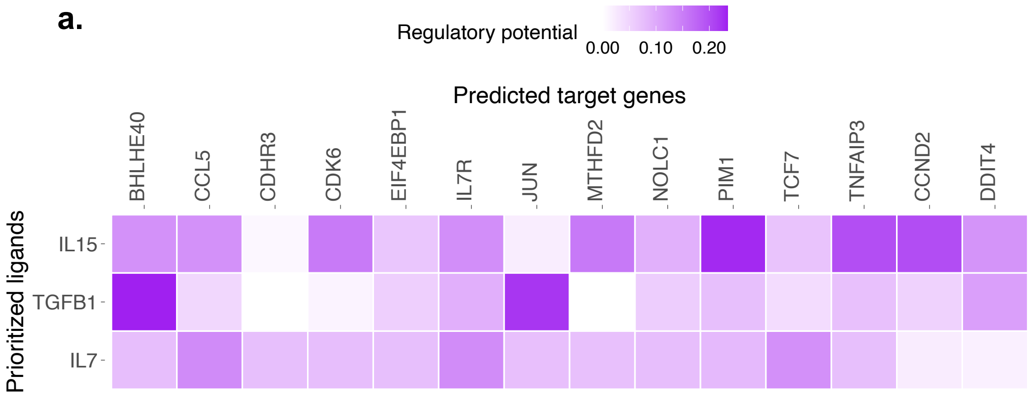

b.

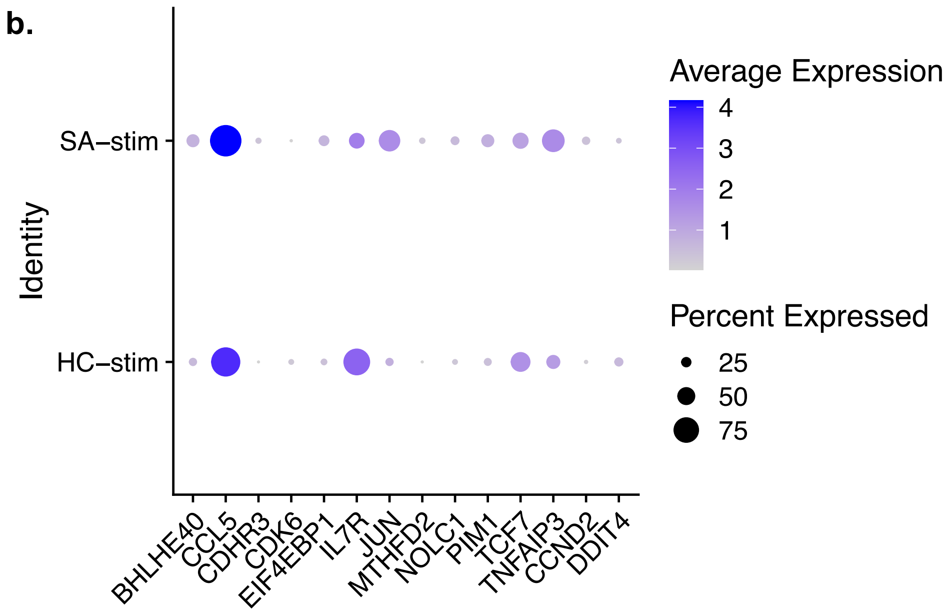

c.

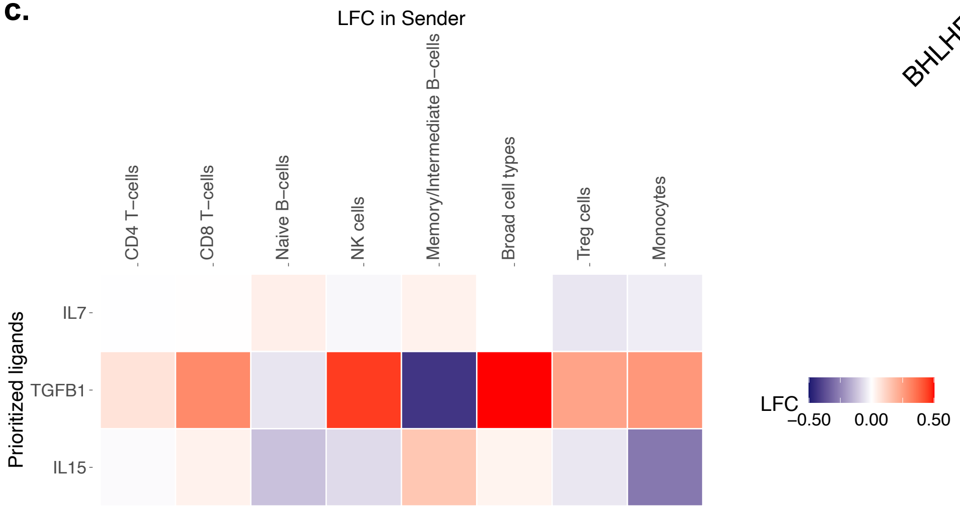

Supplemental Figure 7D. NicheNet cell-cell communication analysis of ligands and receptors in the SA-stim vs HC-stim comparison. (a) Ligand and target gene analysis showing the top expressed ligands (rows) with their inferred target genes (columns). (b) Dot plot showing expression levels of the target genes from (a) between the SA-stim and HC-stim sample groups. (c) Log fold changes of the ligands (rows) in each sender cell type (columns) between SA-stim and HC-stim.

Supplemental Figure 8. TCR γδ-high Population in SA Patients

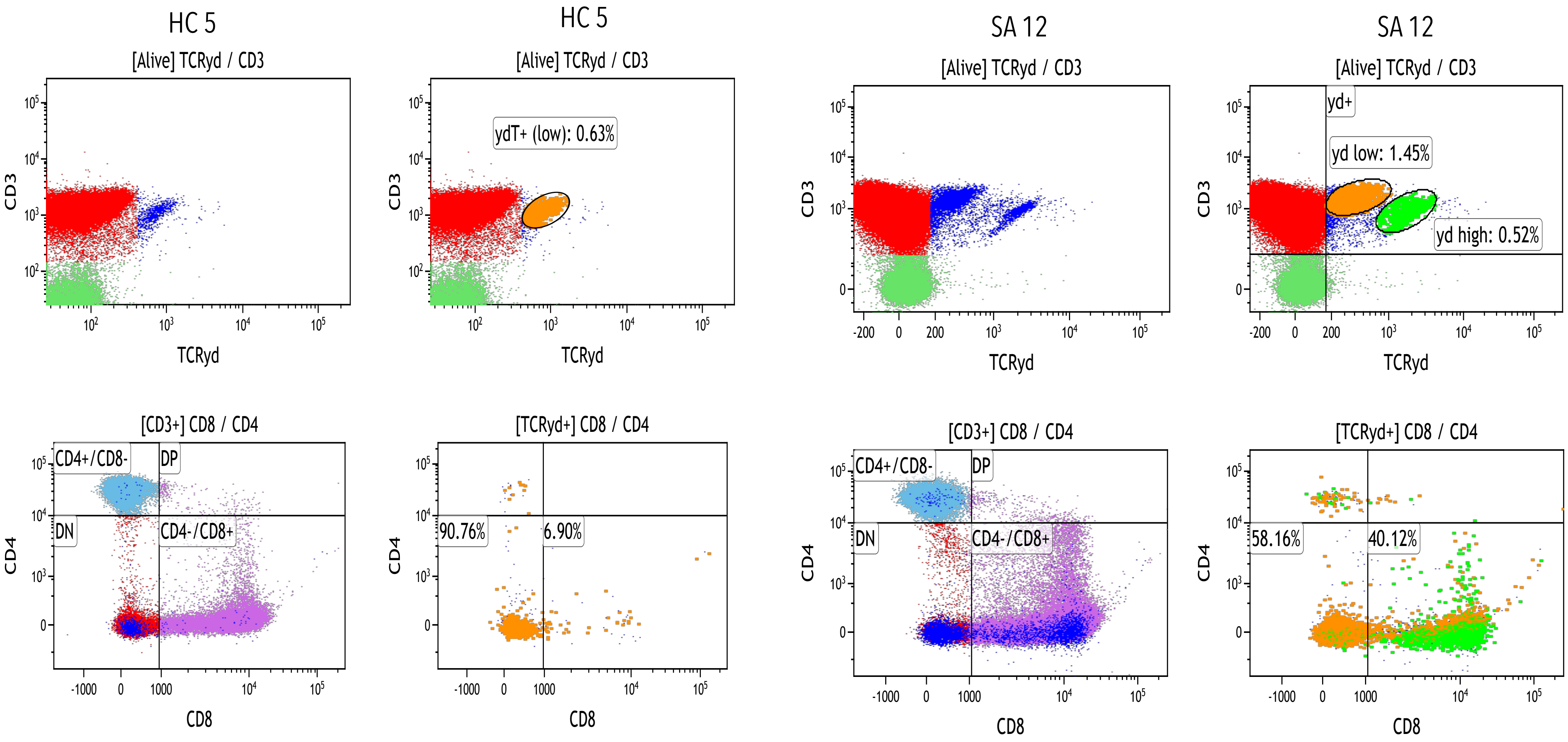

Supplemental Figure 8. TCR γδ-high Population in SA Patients

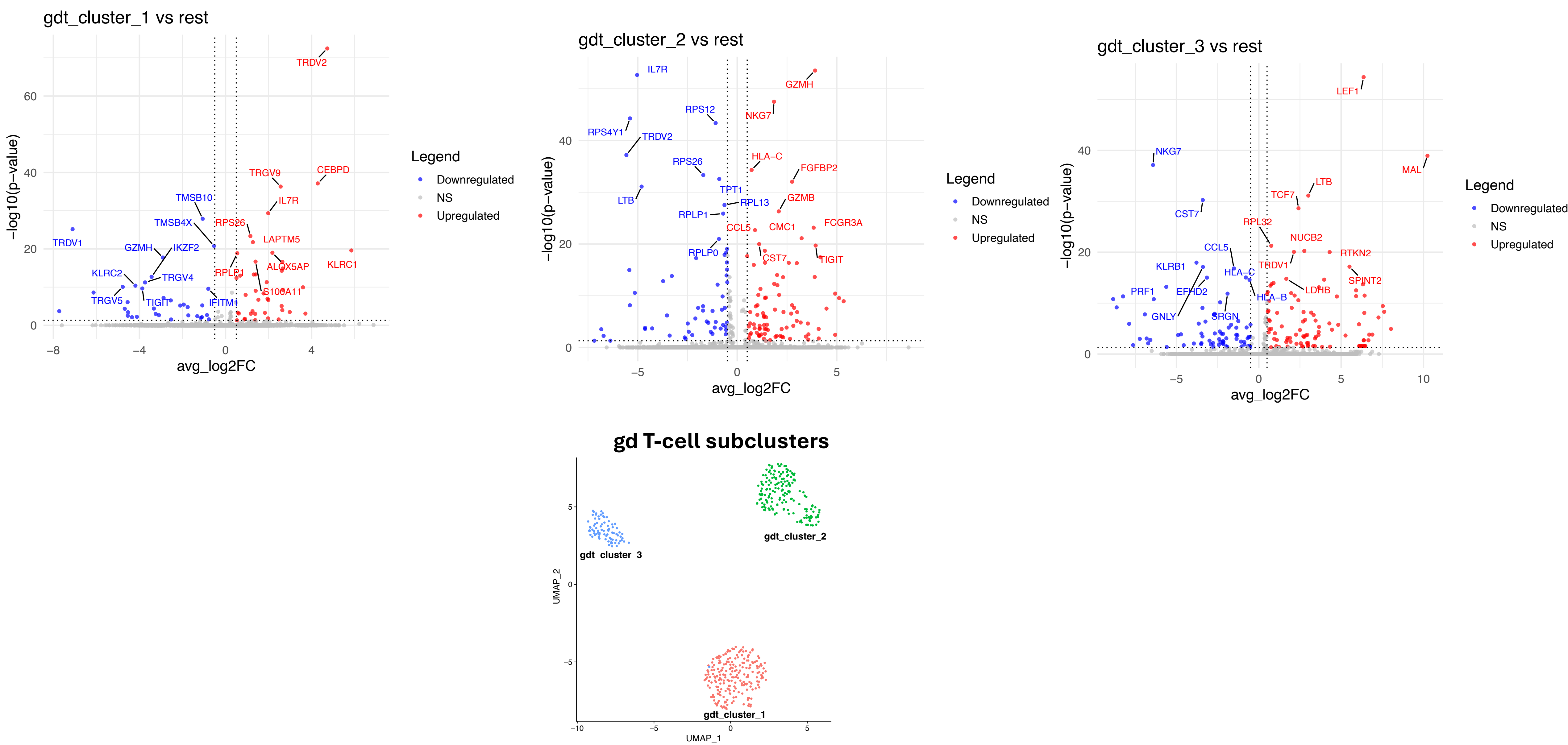

Supplemental Figure 9. Volcano plots of differential gene expression (DEG) analysis of gd T cell subclusters 1-3.

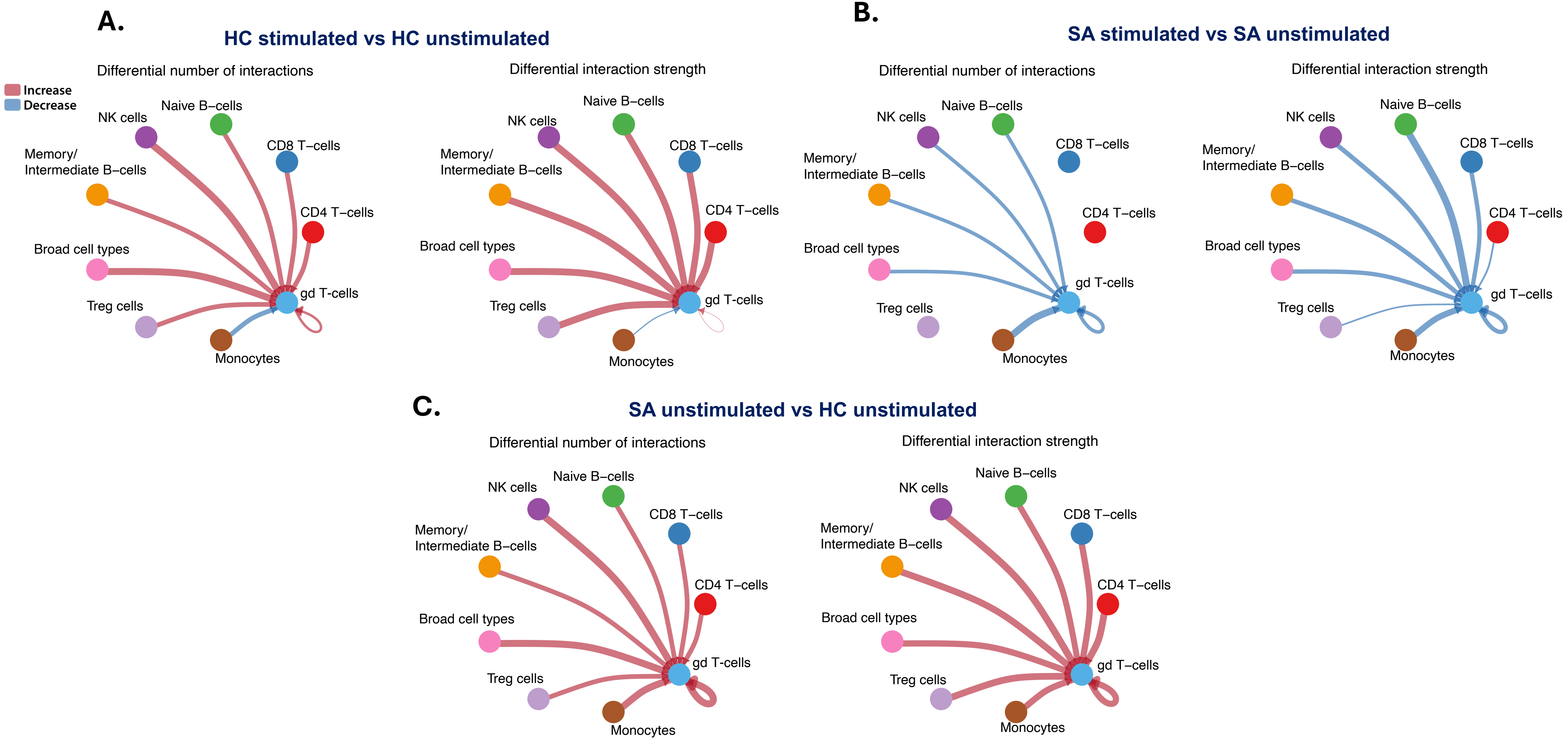

Supplemental Figure 10. CellChat analysis of the cell-cell interactions in PBMC in response to TM stimulation. (A-C) Circle plots displaying the differential number of interactions and interaction strength (red = upregulated, blue = downregulated) in γδ T-cells (blue circle). The direction of the signaling is shown with the arrows and the number of interactions depicted by the width of the arrows. Autocrine activity is shown by the arrow coming from and leading back to the blue γδ T-cell circle. Differential interactions are depicted in the following samples: **(A)** HC-stim vs HC-unstim, **(B)** SA-stim vs SA-unstim, **(C)** SA-unstim vs HC-unstim.

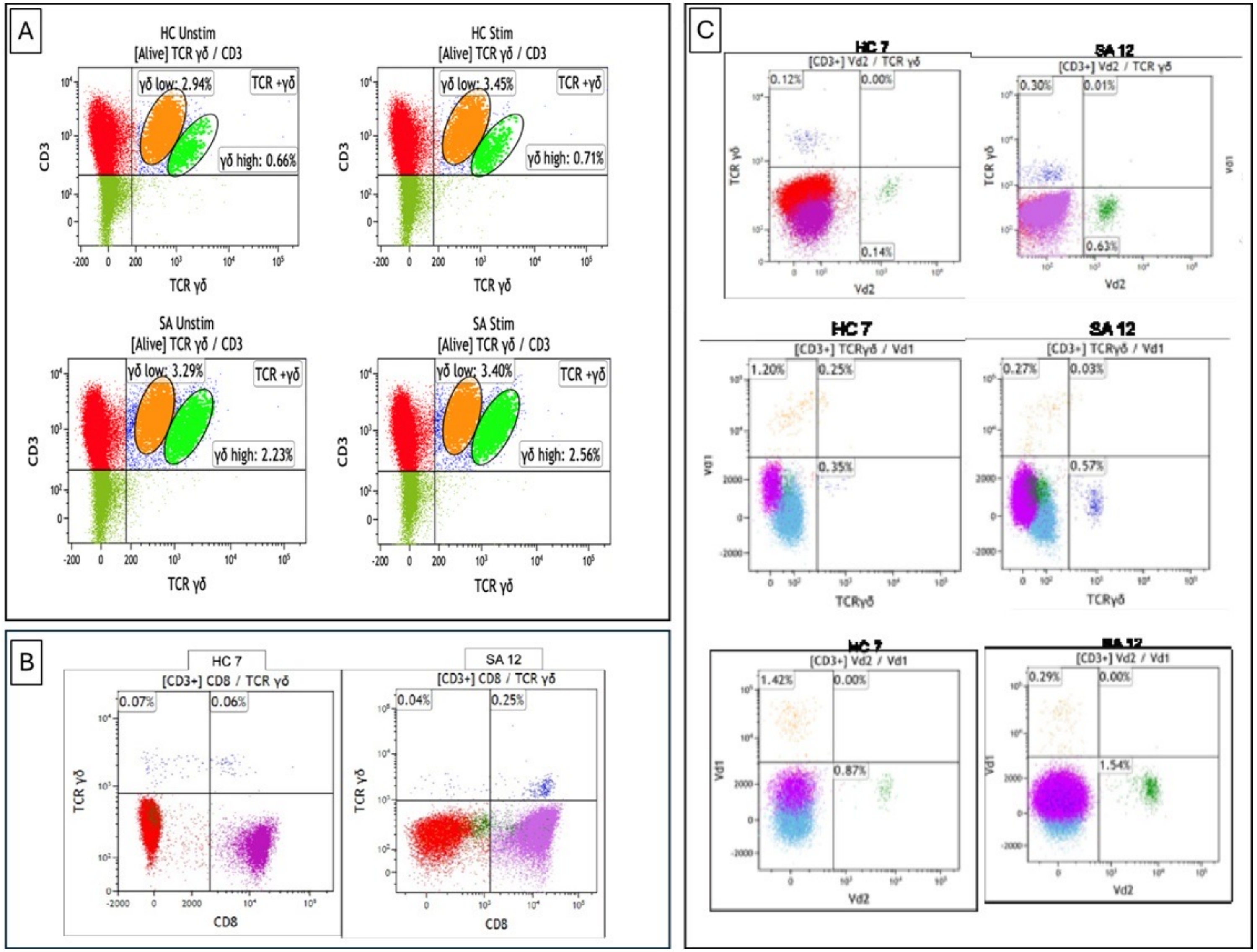

Supplementary Figure 11: Characterization of γδ T-cell Cell clusters in Healthy Control (HC) and Shrimp Allergy (SA) Samples by Flow Cytometric Analysis. **(A)** Distinct γδ TCR^{high} and TCR^{low} T cell populations in shrimp allergic patients from the pooled HC (n=3) and SA (n=2) after TM stimulation. The SA cells show a higher proportion of a γδ TCR^{high} T cell population (2%). **(B)** γδ TCR^{high} population in individual SA patients shows the SA γδ TCR^{high} population is predominantly CD8+. **(C)** Vd1 and Vd2 staining reveals variable delta chain dimorphism within CD8+ cells. Vd2+ and TCR γδ cell populations are higher in SA compared to HC.
